# Fabrication of Alternating Copolymers Based on Cyclopentadithiophene-Benzothiadiazole Dicarboxylic Imide with Reduced Optical Band Gap: Synthesis, Optical, Electrochemical, Thermal, and Structural Properties

**DOI:** 10.3390/polym13010063

**Published:** 2020-12-26

**Authors:** Ary R. Murad, Ahmed Iraqi, Shujahadeen B. Aziz, Sozan N. Abdullah, Mohamad A. Brza, Salah R. Saeed, Rebar T. Abdulwahid

**Affiliations:** 1 Department of Pharmaceutical Chemistry, College of Medical and Applied Sciences, Charmo University, Chamchamal 46023, Iraq; ary.murad@charmouniversity.org; 2 Department of Chemistry, University of Sheffield, Sheffield S3 7HF, UK; a.iraqi@sheffield.ac.uk; 3 Hameed Majid Advanced Polymeric Materials Research Lab., Department of Physics, College of Science, University of Sulaimani, Sulaimani 46001, Iraq; mohamad.brza@gmail.com (M.A.B.); rebar.abdulwahid@univsul.edu.iq (R.T.A.); 4 Department of Civil Engineering, College of Engineering, Komar University of Science and Technology, Sulaimani 46001, Iraq; 5 Department of Chemistry, College of Science, University of Sulaimani, Sulaimani 46001, Iraq; sozan.abdulla@univsul.edu.iq; 6 Department of Manufacturing and Materials Engineering, Faculty of Engineering, International Islamic University of Malaysia, Kuala Lumpur, Gombak 53100, Malaysia; 7 Charmo Research Center, Charmo University, Chamchamal 46023, Iraq; salah.saeed@charmouniversity.org

**Keywords:** conjugated copolymers, cyclopentadithiophene, direct arylation polymerization, band gap study, cyclic voltammetry, UV-vis, XRD study, thermogravimetric analysis (TGA)

## Abstract

A series of alternating copolymers containing cyclopentadithiophene (CPDT) flanked by thienyl moieties as electron-donor units and benzothiadiazole dicarboxylic imide (BTDI) as electron-acceptor units were designed and synthesized for solar cell applications. Different solubilizing side chains, including 2-ethylhexyl chains and *n*-octyl chains were attached to CPDT units, whereas 3,7-dimethyloctyl chains and *n*-octyl chains were anchored to the BTDI moieties. The impact of these substituents on the solubilities, molecular weights, optical and electrochemical properties, and thermal and structural properties of the resulting polymers was investigated. PCPDTDTBTDI-EH, DMO was synthesized via Suzuki polymerization, whereas PCPDTDTBTDI-8, DMO, and PCPDTDTBTDI-EH, 8 were prepared through direct arylation polymerization. PCPDTDTBTDI-8, DMO has the highest number average molecular weight (*M*_n_ = 17,400 g mol^−1^) among all polymers prepared. The PCPDTDTBTDI-8, DMO and PCPDTDTBTDI-8, 8 which have *n*-octyl substituents on their CPDT units have comparable optical band gaps (E_g_ ~ 1.3 eV), which are around 0.1 eV lower than PCPDTDTBTDI-EH, DMO analogues that have 2-ethylhexyl substituents on their CPDT units. The polymers have their HOMO levels between −5.10 and −5.22 eV with PCPDTDTBTDI-EH, DMO having the deepest highest occupied molecular orbital (HOMO) energy level. The lowest unoccupied molecular orbital (LUMO) levels of the polymers are between −3.4 and −3.5 eV. All polymers exhibit good thermal stability with decomposition temperatures surpassing 350 °C. Powder X-ray diffraction (XRD) studies have shown that all polymers have the amorphous nature in solid state.

## 1. Introduction

Cyclopenta [2,1-*b*:3,4-*b*′]dithiophene (CPDT) derivatives are analogous materials to fluorene derivatives, where two thiophenes rather than two phenyl groups are bridged by a carbon atom. These materials have attracted considerable attention [1]. The CPDT unit has a much stronger electron-donating ability than fluorene and hence has stronger orbital mixing with electron-deficient moieties. In addition, donor-acceptor (D-A) copolymers based on the CPDT have higher planarity and lower optical band gaps, which offer enhanced conjugation and stronger intermolecular interactions resulting in high charge carrier mobilities [2,3]. One of the most promising D-A low band gap (E_g_) copolymers containing the CPDT unit is poly[2,6-(4,4-bis(2-ethylhexyl)-4*H*-cyclopenta[2,1-*b*:3,4-*b*′]dithiophene)-*alt*-2,1,3-benzothiadiazole] (PCPDTBT). PCPDTBT is an alternating copolymer comprising the CPDT as the donor moiety and 2,1,3-benzothiadiazole (BT) as an acceptor unit, which was first synthesized using Stille polymerization by Brabec et al. and used in photovoltaic cells [4]. It has an optical band gap (E_g_) of around 1.4 eV with a broad absorption up to 850 nm. PCPDTBT fabricated with PC_71_BM achieved a PCE of 3.5% [5,6]. Bazan and Heeger groups further optimized devices based on PCPDTBT: PC_71_BM and the photovoltaic performance up to 5.5% was recorded [7,8]. Brabec et al. reported a high hole mobility for PCPDTBT of 0.02 cm^2^ V^−1^ s^−1^. Zhang and coworkers synthesized PCPDTBT with *n*-hexyldecyl chains on the CPDT unit and field effect transistor (FET) devices based on this polymer showed a higher hole mobility of 0.17 cm^2^ V^−1^ s^−1^ due to enhanced solubility and increased packing order [9,10]. Recently, a hole mobility as high as 3.3 cm^2^ V^−1^ s^−1^ based on the same polymer has been achieved by Tsao et al. [11]. A series of D-A copolymers were synthesized based on the CPDT as the donor building blocks and different acceptor moieties instead of BT. For instance, Janssen group synthesized new low band gap copolymers, PCPDTBO by copolymerizing CPDT unit with 2,1,3-benzoxadiazole (BO) as an electron acceptor unit [12]. PCPDTBO: PC_61_BM bulk heterojunction (BHJ) solar cell gave the PCE of 2.5%. PCPDTDTBT was synthesized by copolymerizing CPDT flanked by thienyl units as the donor unit with BT as the acceptor unit. BHJ solar cells based on PCPDTDTBT: PC_61_BM showed the PCE of 2.1% [13]. 

Li et al. synthesized a new copolymer, PCPDTDTBTz by copolymerizing CPDT flanked by thienyl units as the electron donor unit with 2,2′-bithiazole (BTz) as an electron acceptor unit [14]. PCPDTDTBTz: PC_61_BM BHJ photovoltaic cells delivered the PCE of 3%. Li and coworkers further synthesized two copolymers, PCPDT_H_DT_H_Tz and PCPDT_H_DT_EH_Tz based on CPDT flanked by thienyl units as the donor units and 1,2,4,5-tetrazine (Tz) as an acceptor unit. PCPDT_H_DT_H_Tz has a molecular weight almost twice than its PCPDT_H_DT_EH_Tz analogue due to less steric hindrance from the linear side chains. PCPDT_H_DT_H_Tz fabricated with PC_71_BM in BHJ solar cell gave a high PCE of 5.53%. However, PCPDT_H_DT_EH_Tz delivered lower PCE of 3.89% [15,16].

Alternating copolymers including CPDT as the strong donor units and thieno[3,4-*c*]pyrrole-4,6-dione (TPD) as the acceptor units were reported by several groups. PCPDT_Oc_TPD_Dd_ was synthesized by Guo et al. via Stille polymerization [17,18]. PCPDT_Oc_TPD_Dd_: PC_71_BM exhibited the PCE higher than 3%. Soon after, Li et al. reported PCPDT_H_TPD_Oc_ with *n*-hexyl chains on CPDT units and *n*-octyl chains on the TPD units. PCPDT_H_TPD_Oc_ blended with PC_71_BM in BHJ photovoltaic cell gave an impressive PCE of 6.41% [19].

Polymers can also be used for application in energy storage devices, such as batteries. Electrode materials are essential for improving the performance of energy storage devices. For example, Li and Fan investigated core-shell structured sulphur composite nanoparticles as a promising cathode material for lithium-sulphur batteries and the authors said thanks to their unique features in suppressing the lithium polysulphides shuttle influence, accommodating the sulphur electrode volume change, and producing abundant electrochemically active sites [20]. Li et al. in another study investigated lithium sulphide (Li_2_S) nanoparticles as the cathode material for lithium-sulphur batteries. The authors described that although Li_2_S could not solve all the issues faced by lithium-sulphur batteries and it might also introduce new problems, it provides new opportunities. As the fully lithiated state of sulphur, Li_2_S provides the prospect of lithium-metal-free anodes and will also reduce the volume expansion problems otherwise happening in the sulphur cathode [21].

## 2. Experimental Section

### 2.1. Materials

All of the starting materials and reagents obtained from Sigma-Aldrich (Gillingham, UK) and Alfa Aesar (Heysham, UK) were utilized without further purification. The majority of the reactions were carried out under argon atmosphere. Anhydrous solvents used for the reactions were obtained from Grubbs solvent purification system within the Sheffield University/Chemistry Department. All the monomers used for preparing the polymers in this article were synthesized according to the procedure part.

### 2.2. Measurements

Here, ^1^H and ^13^C nuclear magnetic resonance (NMR) spectra for the monomers were measured either with a Bruker Avance AV 3HD 400 (400 MHz, Bruker, Berlin, Germany) spectrometer in deuterated chloroform (CDCl_3_), deuterated acetone (CD_3_COCD_3_), or deuterated dimethyl sulphoxide (CD_3_SOCD_3_) as the solvents at room temperature. The ^1^H NMR spectra for the polymers were measured with Bruker AV 3HD 500 (500 MHz, Bruker, Berlin, Germany) in deuterated 1,1,2,2-tetrachloroethane (C_2_D_2_Cl_4_) as the solvent at 100 °C. The chemical shifts were measured in parts per million (ppm). The coupling constants (*J*) are calculated in Hertz (Hz). The ^1^H and ^13^C NMR spectra were analysed using Bruker TopSpin 3.2 software. Elemental analysis (CHN) was performed by either the Perkin Elmer 2400 CHNS/O Series II Elemental Analyser (Horiba, Northampton, UK) or Vario MICRO Cube CHN/S Elemental Analyser (Eltra, Chester, UK) for CHN analysis. Anion analysis (Br, I, and S) was performed by the Schöniger oxygen flask combustion method. Mass spectra for the monomers were recorded on Agilent 7200 accurate mass Q-TOF GC-MS spectrometer (Agilent, Santa Clara, CA, USA). Helium was used as a carrier gas at the rate of 1.2 mL min^−1^, the injection volume was 1.0 μL, and the concentration of measured sample was 5 mg mL^−1^ in CHCl_3_ solvent. The temperature program is between 60 and 320 °C at 10 °C min^−1^. Mass spectra for the monomers were obtained by the electron ionization method (EI). Gel permeation chromatography (GPC) measurements accomplished by Viscotek GPC Max (Malvern Panalytical, Malvern, UK), a waters 410 instrument with a differential refractive index detector, two Polymer Labs PLgel 5μ Mixed C (7.5 × 300 mm) columns and a guard (7.5 × 50 mm). Molecular weights for the polymers were determined by preparing polymer solutions (2.5 mg mL^−1^) using HPLC grade CHCl_3_. The columns were thermostated at 40 °C using CHCl_3_. UV-VIS absorption spectra were measured by SPECORD S600 UV/visible Spectrophotometer (Hach, Düsseldorf, Germany) at room temperature. The absorbance of the polymers was measured in CHCl_3_ solution using quartz cuvettes (light path length = 10 mm) and blank quartz cuvettes including CHCl_3_ was used as a reference. The polymers were coated on quartz substrates from CHCl_3_ solutions (1 mg mL^−1^) and blank quartz substrate was used as a reference. Thermogravimetric analysis (TGA) measurements were recorded by Perkin Elmer (Pyris 1) thermogravimetric Analyser (Eltra, Chester, UK). Platinum pans was used as sample holder and the weight of the measured samples was about (3 mg). Cyclic voltammograms were measured using a Model 263A Potentiostat/Galvanostat—Princeton Applied Research (Artisan Technology Group, Champaign, Indian, United State). A standard three electrode system was used based on a Pt disk working electrode, a silver wire reference electrode (Ag/Ag^+^) inserted in (0.01 M) AgNO_3_ solution in acetonitrile and put it in the electrolyte solution and a Pt wire counter electrode was purged with argon atmosphere during all measurements at room temperature. Tetrabutylammonium perchlorate in acetonitrile (Bu_4_NClO_4_/CH_3_CN) (0.1 M) was used as the electrolyte. Polymer thin films were drop cast onto the Pt disk from polymer solutions in CH_3_Cl (1 mg mL^−1^) and dried under nitrogen prior to measurement. Ferrocene (Fc/Fc^+^) was used as a reference redox system. Powder X-ray diffraction (XRD) for the polymers was measured by Bruker D8 ADVANCE X-ray powder diffractometer (Bruker, Berlin, Germany). Infrared absorption spectra were recorded on ATR Perkin Elmer Rx/FT-IR system (Perkin Elmer, Melville, NY, USA) and Nicolet Model 205 FT-IR spectrometer (Nicolet Instrument, Sainte-Julie, QC, Canada).

### 2.3. Monomers and Polymers Synthesis

Synthesis of 2,5-dibromothiophene (1): Thiophene (25.00 g, 297.12 mmol) in DMF (250 mL) was added to a flask and cooled to −15 °C. To this solution, NBS (110.00 g, 618.04 mmol) in DMF (300 mL) was added dropwise in the dark, and the reaction was stirred overnight at RT. The reaction contents were put into ice and DCM and subsequently extracted with DCM, and the organic phase was washed with deionized H_2_O to a neutral pH. The organic layer was collected and dried over MgSO_4_ and the solvent was concentrated to afford the product, which was purified by vacuum distillation and gave 1 as a yellow oil (59.30 g, 245 mmol, 82% yield) [22]. ^1^H NMR (CDCl_3_, δ): 6.87 (s, 2H). ^13^C NMR (CDCl_3_, δ): 130.4, 111.6. FT-IR (cm^−1^): 3096, 1726, 1516, 1410, 1200. EI-MS (*m*/*z*): 242 [M]^+^. Elemental analysis (%) calculated for C_4_H_2_Br_2_S: C, 19.86; H, 0.83; Br, 66.06, S, 13.25. Found: C, 20.01; H, 0.85; Br, 65.02, S, 11.96.

Synthesis of 2,5-dibromo-3,4-dinitrothiophene (2): Concentrated H_2_SO_4_ (150 mL) and fuming H_2_SO_4_ (150 mL, 20% free SO_3_) were combined in a flask. This flask was cooled to 0 °C and 1 (26.00 g, 107.46 mmol) was added dropwise. Concentrated nitric acid (125 mL) was added dropwise, and the reaction contents were kept under 20 °C. During addition of nitric acid, yellow precipitate formed quickly. The mixture was stirred for 3 h at 20–30 °C. Then, the mixture was poured into ice, and upon melting of the ice, a yellow precipitate was filtrated and washed thoroughly with deionized H_2_O. The product recrystallized from methanol to afford 2 as yellow crystals (32.50 g, 98 mmol, 91% yield) [23]. ^13^C NMR (CDCl_3_, δ): 140.7, 113.4. FT-IR (cm^−1^): 2886, 2851, 2813, 1535, 1497, 1345, 1081. EI-MS (*m*/*z*): 332 [M]^+^. Elemental analysis (%) calculated for C_4_Br_2_N_2_O_4_S: C, 14.47; N, 8.44; S, 9.66; Br, 48.15. Found: C, 14.51; N, 7.91; S, 9.19; Br, 46.57. 

Synthesis of 3′,4′-dinitro-2,2′:5′,2″-terthiophene (3): In a flask, 2 (9.90 g, 29.82 mmol), 2-(tributylstannyl)thiophene (27.82 g, 74.54 mmol) and PdCl_2_(PPh_3_)_2_ (0.45 g, 0.64 mmol) were added. The system degassed under argon and anhydrous toluene (100 mL) was added and heated at 115 °C for 24 h. The flask was cooled to RT and the volatiles were removed to obtain the product, which was purified by column chromatography with gradient (petroleum ether, 0–50% DCM) to obtain an orange solid and the product further purified by recrystallization from methanol to afford 3 as orange crystals (9.10 g, 27 mmol, 90% yield) [24]. ^1^H NMR (CDCl_3_, δ): 7.62 (dd, 2H, J = 1.0 Hz, 5.0 Hz), 7.56 (dd, 2H, J = 1.0 Hz, 4.0 Hz), 7.19 (dd, 2H, J = 4.0 Hz, 5.0 Hz). ^13^C NMR (CDCl_3_, δ): 135.9, 133.9, 131.3, 131.2, 128.4, 128.0. FT-IR (cm^−1^): 3076, 1821, 1528, 1379, 1348, 1299, 1223, 1066. EI-MS (*m*/*z*): 338 [M]^+^. Elemental analysis (%) calculated for C_12_H_6_N_2_O_4_S_3_: C, 42.60; H, 1.79; N, 8.28; S, 28.42. Found: C, 42.49; H, 1.66; N, 8.13; S, 28.16. 

Synthesis of 3′,4′-diamino-2,2′:5,2″-terthiophene (4): EtOH (31 mL) and HCl (62 mL, 35%) were added to 3 (3.00 g, 8.86 mmol) in a flask. To this mixture, anhydrous tin (II) chloride (31.00 g, 163.50 mmol) in ethanol (62 mL) was added and stirred at 30 °C for 24 h. The mixture was cooled to RT and put into cold NaOH. To this mixture, toluene was added and then stirred vigorously and filtered through celite. The product was extracted with toluene and the organic phases were washed with NaCl and subsequently dried over MgSO_4_. The solvent was concentrated to obtain the 4 as a brown solid (2.40 g, 9 mmol, 97% yield) [25]. ^1^H NMR (CDCl_3_, δ): 7.30 (d, 2H, J = 2.0 Hz), 7.27 (s, 2H), 7.09–7.14 (m, 2H), 3.76 (bs, 4H). ^13^C NMR (CDCl_3_, δ): 136.0, 133.6, 127.8, 124.0, 124.0, 110.1. FT-IR (cm^−1^): 3371, 3298, 3224, 3182, 3096, 1631, 1615, 1573, 1528, 1509, 1441, 1336, 1294, 1070. EI-MS (*m*/*z*): 278 [M]^+^. Elemental analysis (%) calculated for C_12_H_10_N_2_S_3_: C, 51.77; H, 3.62; N, 10.06; S, 34.55. Found: C, 51.69; H, 3.54; N, 9.97; S, 34.78. 

Synthesis of 4,6-bis(2-thienyl)-thieno[3,4-c][1,2,5]-thiadiazole (5): Product 4 (1.67 g, 5.99 mmol) was dissolved in dry pyridine (30 mL) in a flask and degassed under argon. To this mixture, *N*-thionylaniline (1.60 g, 11.49 mmol) was added dropwise and chlorotrimethylsilane (4.50 g, 41.42 mmol) was then added dropwise, resulting in a dark blue colour. The reaction contents were stirred for 3 h at RT and then put into DCM. The solution was washed with HCl and with deionized water and then extracted with DCM. The organic phase was dried over anhydrous MgSO_4_ and subsequently filtered. The solvent was evaporated to afford the product, which purified via chromatography with DCM to afford 5 as blue crystals (1.72 g, 6 mmol, 93% yield) [26]. ^1^H NMR (CDCl_3_, δ): 7.59 (dd, 2H, J = 1.0 Hz, 3.5 Hz), 7.34 (dd, 2H, J = 1.0 Hz, 5.0 Hz), 7.12 (dd, 2H, J = 3.5 Hz, 5.0 Hz). ^13^C NMR (CDCl_3_, δ): 156.3, 135.0, 128.2, 125.4, 124.3, 112.4. FT-IR (cm^−1^): 3102, 3073, 1797, 1525, 1483, 1365, 1223, 1137, 1047. EI-MS (*m*/*z*): 306 [M]^+^. Elemental analysis (%) calculated for C_12_H_6_N_2_S_4_: C, 47.04; H, 1.97; N, 9.14; S, 41.85. Found: C, 47.25; H, 2.18; N, 8.83; S, 39.16. 

Synthesis of 4,7-di(thien-2-yl)-2,1,3-benzothiadiazole-5,6-dimethyl ester (6): 5 (1.86 g, 6.06 mmol) and dimethyl acetylenedicarboxylate (1.73 g, 12.17 mmol) were combined in a flask. The system was evacuated and refilled with argon for three cycles before anhydrous xylene (40 mL) was added. The reaction contents were refluxed for 24 h. The flask was cooled to RT and the solvent was removed to afford the product, which was purified by column chromatography with gradient (petroleum ether, 0–50% DCM) to afford 6 as yellow crystals (2.37 g, 6 mmol, 94% yield) [27]. ^1^H NMR (CDCl_3_, δ): 7.62 (dd, 2H, J = 1.0 Hz, 5.0 Hz), 7.44 (dd, 2H, J = 1.0 Hz, 3.5 Hz), 7.22 (dd, 2H, J = 3.5 Hz, 5.0 Hz), 3.78 (s, 6H). ^13^C NMR (CDCl_3_, δ): 168.1, 153.6, 135.1, 132.0, 129.7, 129.0, 127.3, 126.2, 53.1. FT-IR (cm^−1^): 3109, 2975, 2932, 2900, 2865, 2159, 2031, 1971, 1730, 1513, 1460, 1318, 1283, 1198. EI-MS (*m*/*z*): 416 [M]^+^. Elemental analysis (%) calculated for C_18_H_12_N_2_O_4_S_3_: C, 51.91; H, 2.90; N, 6.73; S, 23.09. Found: C, 51.86; H, 2.94; N, 6.61; S, 22.97.

Synthesis of 4,7-di(thien-2-yl)-2,1,3-benzothiadiazole-5,6-dicarboxylic acid (7): Sodium hydroxide (4.00 g, 100.00 mmol) dissolved in deionized water (30 mL) was added to a flask. To this solution, ethanol (200 mL) and 6 (2.27 g, 5.45 mmol) were added and the reaction contents were refluxed for 24 h. The flask was cooled to RT and deionized H_2_O was added. This mixture was cooled to 0 °C and neutralized by HCl to precipitate the product. The precipitate was filtered and subsequently washed with deionized H_2_O. The precipitate was dried under high vacuum to afford 7 as yellow solid (1.80 g, 5 mmol, 85% yield) [28]. ^1^H NMR (CD_3_SOCD_3_, δ): 7.86 (dd, 2H, J = 1.0 Hz, 5.0 Hz), 7.47 (dd, 2H, J = 1.0 Hz, 3.5 Hz), 7.25 (dd, 2H, J = 3.5 Hz, 5.0 Hz). ^13^C NMR (CD_3_SOCD_3_, δ): 168.4, 152.5, 134.8, 133.0, 129.7, 129.3, 127.2, 123.8. FT-IR (cm^−1^): 3106, broad (3300–2600), 2162, 2024, 1971, 1815, 1765, 1705, 1552, 1453, 1386, 1261, 1152, 1020. EI-MS (*m*/*z*): 387 [M-H]^+^. Elemental analysis (%) calculated for C_16_H_8_N_2_O_4_S_3_: C, 49.48; H, 2.08; N, 7.21; S, 24.76. Found: C, 45.33; H, 2.70; N, 6.47; S, 21.35.

Synthesis of 4,7-di(thien-2-yl)-2,1,3-benzothiadiazole-5,6-dicarboxylic anhydride (8): 7 (1.15 g, 2.96 mmol) and anhydrous acetic anhydride (10.00 g, 97.95 mmol) were combined in a flask. The system was evacuated and refilled with argon for three cycles before anhydrous xylene (30 mL) was added. The mixture was heated at 130 °C for 6 h. The mixture was cooled to RT, and the solvent was evaporated to obtain 8 as red solid (1.06 g, 3 mmol, 97% yield) [29]. ^1^H NMR (CDCl_3_, δ): 8.11 (dd, 2H, J = 1.0 Hz, 4.0 Hz), 7.82 (dd, 2H, J = 1.0 Hz, 5.0 Hz), 7.33 (dd, 2H, J = 4.0 Hz, 5.0 Hz). ^13^C NMR (CD_3_SOCD_3_, δ): 162.0, 156.0, 134.3, 132.6, 131.4, 127.8, 127.6, 125.5. FT-IR (cm^−1^): 3131, 3109, 3081, 1808, 1765, 1552, 1453, 1393, 1247, 1152, 1088. EI-MS (*m*/*z*): 370 [M]^+^. Elemental analysis (%) calculated for C_16_H_6_N_2_O_3_S_3_: C, 51.88; H, 1.63; N, 7.56; S, 25.97. Found: C, 52.11; H, 2.00; N, 7.20; S, 24.55.

Synthesis of 3,7-dimethyloctyl bromide (9): Triphenylphosphine (21.10 g, 80.44 mmol) was added to a mixture of 3,7-dimethyloctyl alcohol (12.61 g, 79.69 mmol) and dichloromethane (250 mL) and stirred in a flask. To this mixture, NBS (14.26 g, 80.14 mmol) was added portionwise and stirred at RT for 90 min. The mixture was washed with NaHCO_3_ solution, dried over MgSO_4_, and filtered, and the solvent was evaporated. The substance was stirred in petroleum ether for 1 h at RT and then filtered, and the filtrate was evaporated. The product was purified by chromatography with petroleum ether to yield 9 as colourless oil (23.00 g, 59 mmol, 73% yield) [30]. ^1^H NMR (CDCl_3_, δ): 3.55–3.37 (m, 2H), 1.96–1.83 (m, 1H), 1.77–1.61 (m, 2H), 1.60–1.49 (m, 1H), 1.41–1.24 (m, 3H), 1.22–1.11 (m, 3H), 0.82–0.94 (m, 9H). ^13^C NMR (CDCl_3_, δ): 40.1, 39.2, 36.7, 32.3, 31.7, 28.0, 24.6, 22.7, 22.6, 19.0. FT-IR (cm^−1^): 2953, 2925, 2868, 1464, 1382, 1261, 1173. EI-MS (*m*/*z*): 222.1 [M]^+^. Elemental analysis (%) calculated for C_10_H_21_Br: C, 54.30; H, 9.57; Br, 36.13. Found: C, 55.04; H, 9.53; Br, 34.23.

Synthesis of N-(3,7-dimethyloctyl)phthalimide (10): 9 (4.07 g, 18.40 mmol) and anhydrous DMF (20 mL) were added into a flask. To this mixture, potassium phthalimide (3.75 g, 20.27 mmol) was added, and the reaction contents were heated to 90 °C for 17 h. The mixture was cooled to RT and put in deionized H_2_O, and the product was subsequently extracted with DCM. The organic extracts were combined, and then washed with KOH and deionized water. The organic phase was dried over MgSO_4_, and the solvent was evaporated to obtain the product, which was purified via chromatography with DCM to yield 10 as colourless oil (5.29 g, 18 mmol, 91% yield) [31]. ^1^H NMR (CDCl_3_, δ): 7.85 (dd, 2H, J = 3.0 Hz, 5.5 Hz), 7.72 (dd, 2H, J = 3.0 Hz, 5.5 Hz), 3.80–3.66 (m, 2H), 1.77–1.66 (m, 1H), 1.53–1.43 (m, 3H), 1.41–1.25 (m, 3H), 1.20–1.11 (m, 3H), 0.98 (d, 3H, J = 6.5 Hz), 0.87 (d, 6H, J = 7.0 Hz). ^13^C NMR (CDCl_3_, δ): 168.4, 133.8, 132.2, 123.1, 39.2, 37.0, 36.3, 35.5, 30.7, 27.9, 24.5, 22.7, 22.6, 19.4. FT-IR (cm^−1^): 2953, 2925, 2868, 1772, 1706, 1616, 1469, 1398, 1267, 1189, 1055. EI-MS (*m*/*z*): 288.2 [MH]^+^. Elemental analysis (%) calculated for C_18_H_25_NO_2_: C, 75.22; H, 8.77; N, 4.87. Found: C, 72.17; H, 8.62; N, 4.43.

Synthesis of 3,7-dimethyl-1-octanamine (11): 10 (6.03 g, 20.98 mmol), hydrazine hydrate (4.0 mL, 65.0 mmol, 51%), and methanol (100 mL) were combined in a flask. The reaction contents were refluxed until the starting material disappeared. Upon completion, excess HCl was added and the mixture was refluxed for 1 h and then cooled to RT. The precipitate was filtered and washed with water. The methanol was concentrated and the residue was diluted with dichloromethane. The organic layer was washed with KOH, and the product was extracted with dichloromethane. The organic phase was washed with NaCl and dried over MgSO_4_, and the solvent was concentrated to yield 11 as a brown oil (2.85 g, 18 mmol, 86% yield) [32]. ^1^H NMR (CDCl_3_, δ): 2.82–2.62 (m, 2H), 1.60–1.43 (m, 3H), 1.35–1.22 (m, 4H), 1.20–1.06 (m, 3H), 0.88 (dd, 9H, J = 2.0 Hz, 6.5 Hz). ^13^C NMR (CDCl_3_, δ): 41.1, 40.1, 39.3, 37.3, 30.5, 28.0, 24.7, 22.7, 22.6, 19.6. FT-IR (cm^−1^): 3521, 3375, 3219, 3021, 2953, 2925, 2868, 2155, 2028, 1978, 1598, 1464, 1382, 1166, 1063. EI-MS (*m*/*z*): 157.2 [M]^+^. Elemental analysis (%) calculated for C_10_H_23_N: C, 76.36; H, 14.74; N, 8.90. Found: C, 71.74; H, 13.51; N, 7.71. 

Synthesis of 4,7-di(thien-2-yl)-2,1,3-benzothiadiazole-5,6-*N*-(3,7-dimethyloctyl)dicarboxylic imide (M1): 8 (1.00 g, 2.69 mmol), acetic acid (50 mL, 100%), and 11 (0.88 g, 5.59 mmol) were combined in a flask. The system was evacuated, refilled with argon for three cycles, and heated overnight at 110 °C. The mixture was cooled to RT, and then, acetic anhydride (20 mL) was added and heated at 110 °C for 6 h. The mixture was cooled to RT, and the solvent was concentrated to yield the product, which was purified by chromatography with 60:10, petroleum ether: ethyl acetate to afford M1 as an orange solid (1.15 g, 2.3 mmol, 84% yield) [29]. ^1^H NMR (CDCl_3_, δ): 7.91 (dd, 2H, J = 1.0 Hz, 3.5 Hz), 7.73 (dd, 2H, J = 1.0 Hz, 5.0 Hz), 7.30 (dd, 2H, J = 3.5 Hz, 5.0 Hz), 3.84–3.70 (m, 2H), 1.78–1.65 (m, 1H), 1.55–1.43 (m, 3H), 1.39–1.22 (m, 3H), 1.20–1.08 (m, 3H), 0.97 (d, 3H, J = 6.0 Hz), 0.86 (d, 6H, J = 6.0 Hz). ^13^C NMR (CDCl_3_, δ): 165.7, 156.5, 133.1, 131.5, 130.2, 127.0, 126.9, 126.7, 39.2, 37.2, 37.0, 35.2, 31.0, 27.9, 24.6, 22.7, 22.6, 19.4. FT-IR (cm^−1^): 3439, 3102, 3074, 2953, 2925, 2865, 1804, 1751, 1694, 1549, 1453, 1364, 1226, 1162, 1056. EI-MS (*m*/*z*): 510.1 [MH]^+^. Elemental analysis (%) calculated for C_26_H_27_N_3_O_2_S_3_: C, 61.27; H, 5.34; N, 8.24; S, 18.87. Found: C, 61.59; H, 5.56; N, 7.94; S, 16.79.

Synthesis of 4,7-di(thien-2-yl)-2,1,3-benzothiadiazole-5,6-*N*-octyl-dicarboxylic imide (M2): M2 was prepared followed by the same procedure for synthesis of M1 except *N*-octylamine (1.20 g, 9.28 mmol) was used. M2 was obtained as an orange solid (1.20 g, 2.5 mmol, 93% yield) [29]. ^1^H NMR (CDCl_3_, δ): 7.91 (dd, 2H, J = 1.0 Hz, 3.5 Hz), 7.73 (dd, 2H, J = 1.0 Hz, 5.0 Hz), 7.30 (dd, 2H, J = 3.5 Hz, 5.0 Hz), 3.74 (t, 2H, J = 7.5 Hz), 1.65–1.76 (m, 2H), 1.23–1.41 (m, 10H), 0.88 (t, 3H, J = 7.0 Hz). ^13^C NMR (CDCl_3_, δ): 165.8, 156.5, 133.1, 131.5, 130.2, 127.1, 126.9, 126.7, 39.0, 31.8, 29.1, 28.2, 27.0, 22.7, 14.0. FT-IR (cm^−1^): 3443, 3102, 3070, 2918, 2854, 1808, 1754, 1694, 1556, 1457, 1364, 1226, 1169, 1098. EI-MS (*m*/*z*): 481.1 [M]^+^. Elemental analysis (%) calculated for C_24_H_23_N_3_O_2_S_3_: C, 59.85; H, 4.81; N, 8.72; S, 19.97. Found: C, 59.91; H, 4.93; N, 8.70; S, 20.72.

Synthesis of 4,7-di(5-bromo-thien-2-yl)-2,1,3-benzothiadiazole-5,6-*N*-(3,7-dimethyloctyl)dicarboxylic imide (M3): M1 (1.00 g, 1.96 mmol) and THF (100 mL) were combined in a flask. To this mixture, NBS (1.74 g, 9.77 mmol) was added and stirred overnight at RT in the dark. The solvent was evaporated to obtain the product as red solid, and it was subsequently washed with cold CH_3_OH, filtered, and dried. The product was purified via chromatography with DCM to yield M3 as red solid (1.28 g, 2 mmol, 98% yield) [28]. ^1^H NMR (CDCl_3_, δ): 7.80 (d, 2H, J = 4.0 Hz), 7.24 (d, 2H, J = 4.0 Hz), 3.70–3.84 (m, 2H), 1.78–1.66 (m, 1H), 1.54–1.44 (m, 3H), 1.41–1.22 (m, 3H), 1.20–1.11 (m, 3H), 0.98 (d, 3H, J = 6.0 Hz), 0.87 (d, 6H, J = 6.5 Hz). ^13^C NMR (CDCl_3_, δ): 165.6, 155.9, 134.1, 133.0, 129.8, 126.4, 125.8, 118.7, 39.2, 37.3, 37.0, 35.2, 31.0, 27.9, 24.6, 22.7, 22.6, 19.4. FT-IR (cm^−1^): 3429, 3120, 2957, 2918, 2865, 1747, 1691, 1563, 1460, 1364, 1283, 1073. EI-MS (*m*/*z*): 666.9 [M]^+^. Elemental analysis (%) calculated for C_26_H_25_Br_2_N_3_O_2_S_3_: C, 46.78; H, 3.78; Br, 23.94; N, 6.30; S, 14.41. Found: C, 46.61; H, 3.61; Br, 23.95; N, 6.29; S, 14.64.

Synthesis of bis(3-thienyl)methanol (12): In a flask, 3-bromothiophene (19.56 g, 119.97 mmol) was added and then degassed under argon before anhydrous Et_2_O (150 mL) was added. The flask was cooled to −78 °C and *n*-BuLi (48.00 mL, 120.00 mmol) was added dropwise and stirred for 4 h. To this mixture, thiophene-3-carboxaldehyde (13.44 g, 119.83 mmol) was added dropwise. The reaction contents were stirred at −78 °C for 3 h and the temperature was raised to RT and stirred overnight. NH_4_Cl solution was added and the product extracted with CHCl_3_. The organic layer was separated and washed with NaCl solution. The combined organic layers were dried over MgSO_4_ and filtered, and the solvent was evaporated to yield the product. It was purified via column chromatography using petroleum ether: ethyl acetate (80:20) to obtain 12 as yellow oil (20.00 g, 102 mmol, 85% yield) [33]. ^1^H NMR (CDCl_3_, δ): 7.32 (dd, 2H, *J* = 3.0 Hz, 5.0 Hz), 7.25–7.23 (m, 2H), 7.07 (dd, 2H, *J* = 1.0 Hz, J = 5.0 Hz), 5.99 (d, 1H, *J* = 4.5 Hz), 2.22 (d, 1H, *J* = 4.5 Hz). ^13^C NMR (CDCl_3_, δ): 143.0, 126.8, 126.0, 122.4, 73.0. FT-IR (cm^−1^): 3507–3131 (broad), 3099, 3081, 2900, 2865, 2109, 1410, 1304, 1283, 1137, 1077. EI-MS (*m*/*z*): 196.0 [M]^+^. Elemental analysis (%) calculated for C_9_H_8_OS_2_: C, 55.07; H, 4.11; S, 32.67. Found: C, 56.68; H, 3.64; S, 34.12. 

Synthesis of bis(2-iodo-3-thienyl)methanol (13): In a flask, 12 (10.00 g, 50.94 mmol) was added and then degassed under argon before anhydrous Et_2_O (50 mL) was added. The flask was cooled to −78 °C and *n*-BuLi (62.5 mL, 156.25 mmol) was added dropwise at this temperature during 2 h and stirred for 2 h. The reaction contents were stirred at RT for 2 h. The mixture was cooled to −78 °C and subsequently iodine (42.70 g, 168.24 mmol) dissolved in anhydrous Et_2_O (250 mL) was added dropwise. The reaction was stirred overnight at RT. Sodium thiosulphate solution was added, and the product was extracted with Et_2_O. The organic layers were separated and dried over MgSO_4_ and then filtered. The solvent was concentrated to afford the product. It was purified via chromatography using petroleum ether: DCM (70:30) to afford 13 as cream-coloured crystals (18.00 g, 40 mmol, 79% yield) [34]. ^1^H NMR (CDCl_3_, δ): 7.46 (dd, 2H, *J* = 0.5 Hz, 5.5 Hz), 6.96 (d, 2H, *J* = 5.5 Hz), 5.80 (d, 1H, *J* = 3.5 Hz), 2.26 (d, 1H, *J* = 3.5 Hz). ^13^C NMR (CDCl_3_, δ): 146.7, 131.5, 126.9, 75.3, 71.8. FT-IR (cm^−1^): 3507–3131 (broad), 3095, 3085, 2911, 1772, 1747, 1609, 1517, 1400, 1219, 1091. EI-MS (*m*/*z*): 447.8 [M]^+^. Elemental analysis (%) calculated for C_9_H_6_I_2_OS_2_: C, 24.13; H, 1.35; S, 14.31; I, 56.64. Found: C, 24.94; H, 1.28; S, 13.82; I, 57.17.

Synthesis of bis(2-iodo-3-thienyl)ketone (14): 13 (6.64 g, 14.81 mmol) and DCM (150 mL) were combined in a flask. To this mixture, pyridinium chlorochromate (4.79 g, 22.22 mmol) was added and stirred at RT for 24 h. The whole mixture was passed through flash chromatography using DCM to afford 14 as yellow crystals (6.40 g, 14 mmol, 97% yield) [32]. ^1^H NMR (CDCl_3_, δ): 7.49 (d, 2H, *J* = 5.5 Hz), 7.08 (d, 2H, *J* = 5.5 Hz). ^13^C NMR (CDCl_3_, δ): 185.6, 143.2, 131.7, 129.8, 81.3. FT-IR (cm^−1^): 3102, 3077, 1648, 1503, 1396, 1233, 1194, 1063. EI-MS (*m*/*z*): 445.8 [M]^+^. Elemental analysis (%) calculated for C_9_H_4_I_2_OS_2_: C, 24.23; H, 0.90; S, 14.37; I, 56.90. Found: C, 24.30; H, 0.75; S, 14.57, I, 57.09.

Synthesis of *4H*-cyclopenta[2,1-*b*:3,4-*b*′]dithiophen-4-one (15): 14 (3.50 g, 7.84 mmol) and Cu powder (1.49 g, 23.44 mmol) was combined in a flask. The mixture was evacuated and refilled with argon for three cycles before anhydrous DMF (25 mL) was added and heated at 125 °C for 3 h. The reaction contents were cooled to RT, dissolved in toluene, and then filtered. NaHCO_3_ solution was added to the filtrate and the mixture was extracted with toluene, and the organic phases were combined and washed with deionized H_2_O several times until became neutral. The organic phase was dried over anhydrous MgSO_4_ and filtered, and the solvent was evaporated to afford the product, which recrystallized from isopropanol to yield 15 as purple crystals (1.47 g, 8 mmol, 98% yield) [35]. ^1^H NMR (CDCl_3_, δ): 7.06 (d, 2H, *J* = 5.0 Hz), 7.01 (d, 2H, *J* = 5.0 Hz). ^13^C NMR (CDCl_3_, δ): 182.7, 149.2, 142.5, 127.2, 121.8. FT-IR (cm^−1^): 3386, 3102, 3085, 1705, 1354, 1233, 1081. EI-MS (*m*/*z*): 192.0 [M]^+^. Elemental analysis (%) calculated for C_9_H_4_OS_2_: C, 56.23; H, 2.10; S, 33.35. Found: C, 55.94; H, 2.05; S, 33.13. 

Synthesis of *4H*-cyclopenta[2,1-*b*:3,4-*b*′]dithiophene (16): 15 (2.47 g, 12.84 mmol), potassium hydroxide (2.47 g, 44.02 mmol) and hydrazine hydrate (15 mL, 64%) were combined in a flask. The system was evacuated and refilled with argon for three cycles before triethylene glycol (247 mL) was added and heated at 180 °C for 17 h. The flask was cooled to RT and deionized H_2_O was added, and the product was extracted with diethyl ether. The organic layer was washed with NH_4_Cl solution. The organic layer was dried over MgSO_4_ and filtered, and the solvent was concentrated to obtain the product. It was purified via chromatography with petroleum ether to afford 16 as white crystals (1.83 g, 10 mmol, 80% yield) [36]. ^1^H NMR (CDCl_3_, δ): 7.20 (d, 2H, *J* = 5.0 Hz), 7.11 (d, 2H, *J* = 5.0 Hz), 3.56 (s, 2H). ^13^C NMR (CDCl_3_, δ): 149.7, 138.7, 124.5, 123.0, 31.8. FT-IR (cm^−1^): 3095, 3077, 2897, 2765, 1481, 1389, 1251, 1159, 1088. EI-MS (*m*/*z*): 178.0 [M]^+^. Elemental analysis (%) calculated for C_9_H_6_S_2_: C, 60.64; H, 3.39; S, 35.97. Found: C, 60.46; H, 3.39; S, 35.95.

Synthesis of 4,4-bis(2-ethylhexyl)cyclopenta[2,1-*b*:3,4-*b*′]dithiophene (17): 16 (0.25 g, 1.40 mmol), 2-ethylhexyl bromide (0.65 g, 3.36 mmol), and NaI (0.02 g, 0.13 mmol) were combined in a flask. The system was purged with three vacuum/argon cycles before anhydrous DMSO (8.5 mL) was added and cooled to 0 °C. To this mixture, potassium hydroxide (0.31 g, 5.61 mmol) was added and stirred for 17 h at RT. Deionized H_2_O was added to the mixture and extracted with *n*-hexane. The organic phase was separated, dried over anhydrous MgSO_4_, and filtered. The solvent was evaporated to obtain the product. It was purified using chromatography with petroleum ether to afford 17 as yellow oil (0.50 g, 1 mmol, 89% yield) [37]. ^1^H NMR (CDCl_3_, δ): 7.13 (d, 2H, *J* = 5.0 Hz), 6.96–6.92 (m, 2H), 1.96–1.82 (m, 4H), 1.10–0.83 (m, 18H), 0.77 (t, 6H, *J* = 7.0 Hz), 0.60 (t, 6H, *J* = 7.0 Hz). ^13^C NMR (CDCl_3_, δ): 157.6 (t, *J* = 4.0 Hz), 136.8, 124.0, 122.3 (t, 6H, *J* = 4.0 Hz), 53.2, 43.2, 35.0, 34.1, 28.6, 27.3, 22.7, 14.1, 10.6. FT-IR (cm^−1^): 2953, 2921, 2858, 1460, 1379, 1081. EI-MS (*m*/*z*): 402.2 [M]^+^. Elemental analysis (%) calculated for C_25_H_38_S_2_: C, 74.57; H, 9.51; S, 15.92. Found: C, 74.20; H, 9.14; S, 15.38.

Synthesis of 4,4-dioctylcyclopenta[2,1-*b*:3,4-*b*′]dithiophene (18): 18 was prepared following the same procedure for synthesis of 17. Here, 16 (0.65 g, 3.64 mmol), 1-bromooctane (1.25 mL, 8.22 mmol), potassium iodide (0.06 g, 0.38 mmol), anhydrous DMSO (20 mL), and potassium hydroxide (0.81 g, 14.43 mmol) were used. Product 18 was obtained as a yellow oil (1.33 g, 3 mmol, 91% yield) [37]. ^1^H NMR (CDCl_3_, δ): 7.16 (d, 2H, *J* = 5.0 Hz,); 6.95 (d, 2H, *J* = 5.0 Hz,), 1.93–1.80 (m, 4H), 1.37–1.09 (m, 20H), 1.04–0.91 (m, 4H), 0.87 (t, 6H, *J* = 7.0 Hz,). ^13^C NMR (CDCl_3_, δ): 158.1, 136.5, 124.4, 121.7, 53.3, 37.7, 31.8, 30.0, 29.4, 29.3, 24.5, 22.6, 14.1. FT-IR (cm^−1^): 2957, 2921, 2850, 1457, 1379, 1081. EI-MS (*m*/*z*): 402.2 [M]^+^. Elemental analysis (%) calculated for C_25_H_38_S_2_: C, 74.57; H, 9.51; S, 15.92. Found: C, 72.96; H, 9.20; S, 14.56.

Synthesis of 4,4-bis(2-ethylhexyl)-2,6-dibromocyclopenta[2,1-*b*:3,4-*b*′]dithiophene (M4): 17 (1.07 g, 2.65 mmol) was dissolved in DMF (25 mL) in a flask. To this mixture, NBS (0.94 g, 5.28 mmol) was added and stirred at RT for 18 h in the dark. Deionized water was added to the mixture and extracted with Et_2_O. The organic phase was washed with deionized H_2_O until the mixture became neutral. The organic layer was separated, dried over MgSO_4_, and filtered. The solvent was concentrated to obtain the product. It was purified using chromatography with petroleum ether to afford M4 as yellow oil (1.30 g, 2 mmol, 88% yield) [38]. ^1^H NMR (CDCl_3_, δ): 6.95 (t, 2H, *J* = 3.5 Hz), 1.92–1.75 (m, 4H), 1.13–0.84 (m, 18H), 0.80 (t, 6H, *J* = 7.0 Hz), 0.64 (t, 6H, *J* = 7.5 Hz). ^13^C NMR (CDCl_3_, δ): 155.5 (t, *J* = 6.5 Hz), 136.6 (t, *J* = 3.5 Hz), 125.2 (t, *J* = 10.5 Hz), 110.7 (t, *J* = 5.0 Hz), 55.0, 43.0, 35.1, 34.0, 28.5, 27.4, 22.8, 14.1, 10.7. FT-IR (cm^−1^): 2952, 2921, 2853, 1455, 1366, 1174. EI-MS (*m*/*z*): 560.1 [M]^+^. Elemental analysis (%) calculated for C_25_H_36_Br_2_S_2_: C, 53.57; H, 6.47; S, 11.44; Br, 28.51. Found: C, 53.44; H, 6.27; S, 11.50; Br, 28.41.

Synthesis of 4,4-dioctyl-2,6-dibromocyclopenta[2,1-*b*:3,4-*b*′]dithiophene (M5): M5 was prepared following the same procedure for synthesis of M4. Here, 18 (1.23 g, 3.05 mmol), DMF (30 mL), and NBS (1.08 g, 6.06 mmol) were used. M5 was obtained as a green oil (1.48 g, 3 mmol, 87% yield) [38]. ^1^H NMR (CDCl_3_, δ): 6.94 (s, 2H), 1.82–1.71 (m, 4H), 1.36–1.10 (m, 20H), 0.97–0.89 (m, 4H), 0.87 (t, 6H, *J* = 7.0 Hz). ^13^C NMR (CDCl_3_, δ): 156.0, 136.3, 124.6, 111.1, 55.0, 37.5, 31.8, 30.0, 29.3, 29.3, 24.4, 22.6, 14.1. FT-IR (cm^−1^): 2926, 2855, 1457, 1368. EI-MS (*m*/*z*): 560 [M]^+^. Elemental analysis (%) calculated for C_25_H_36_Br_2_S_2_: C, 53.57; H, 6.47; S, 11.44; Br, 28.51. Found: C, 54.00; H, 6.42; S, 11.19; Br, 26.83.

Synthesis of 4,4-bis(2-ethylhexyl)-2,6-bis(4,4,5,5-tetramethyl-1,3,2-dioxaboralan-2-yl)cyclopenta[2,1-*b*:3,4-*b*′]dithiophene (M6): M4 (0.38 g, 0.67 mmol), potassium acetate (0.39 g, 3.97 mmol), bis(pinacolato)diboron (0.51 g, 2.00 mmol), and Pd(dppf)Cl_2_(0.026 g, 0.033 mmol, 5 mol%) were combined in a flask, and then, the system was degassed under argon. To this mixture, anhydrous DMF (30 mL) was wadded and heated at 100 °C for 48 h. The flask was cooled to RT. The product was extracted with Et_2_O, and organic phases were washed with deionized H_2_O. The organic layers were separated and dried over MgSO_4_ and filtered, and the solvent was evaporated to obtain M6 as a red sticky oil (0.25 g, 0.4 mmol, 57% yield) [39]. ^1^H NMR (CDCl_3_, δ): 7.46 (t, 2H, *J* = 6.5 Hz), 1.96–1.78 (m, 4H), 1.36 (s, 24H), 1.10–0.81 (m, 18H), 0.79–0.71 (m, 6H), 0.69–0.55 (m, 6H). ^13^C NMR (CDCl_3_, δ): 161.0, 144.1, 131.9, 84.2, 52.7, 43.4, 35.1, 34.1, 31.8, 28.5, 27.3, 25.0, 24.8, 22.8, 14.1, 10.6. FT-IR (cm^−1^): 2962, 2923, 2862, 1375, 1264, 1139, 1121. EI-MS (*m*/*z*): 654.4 [M]^+^. Elemental analysis (%) calculated for C_37_H_60_B_2_O_4_S_2_: C, 67.89; H, 9.24; S, 9.79. Found: C, 66.36; H, 8.92; S, 7.78.

Synthesis of poly[4,4-bis(2-ethylhexyl)-2,6-cyclopenta[2,1-*b*:3,4-*b*′]dithiophene-*alt*-5,5-(4′,7′-bis(2-thienyl)-2′,1′,3′-benzothiadiazole-5,6-*N*-(3,7-dimethyloctyl) dicarboxylic imide)] (PCPDTDTBTDI-EH, DMO): M6 (147.1 mg, 0.224 mmol) and M3 (150 mg, 0.224 mmol) were added to a flask and degassed under argon. Anhydrous THF (10 mL) followed by sodium hydrogen carbonate solution (2.5 mL, 5% wt, degassed) was added and the system was degassed again. To this mixture, Pd(OAc)_2_ (3.7 mg, 0.0168 mmol) and P(*o*-tol)_3_ (10.2 mg, 0.0336 mmol) were added, degassed, and heated at 90 °C for 30 h. The flask was cooled to RT, the polymer was dissolved in CHCl_3_ (200 mL), an NH_4_OH solution (50 mL, 35% in H_2_O) was added, and the mixture was stirred overnight. The organic phase was separated and washed with deionized H_2_O. The organic phase was concentrated to around (50 mL) and put into methanol (300 mL) and stirred overnight. The mixture was filtered, and the polymer was cleaned using Soxhlet extraction with methanol (300 mL), acetone (300 mL), hexane (300 mL), and then toluene (300 mL). The toluene fraction was concentrated (to around 50 mL) and then put into methanol (300 mL). The mixture was stirred overnight, and the pure polymer was recovered by filtration to afford PCPDTDTBTDI-EH, DMO as dark green powders (16 mg, 0.02 mmol, 8% yield) [40]. GPC: toluene fraction, *M*_n_ = 5200 g mol^−1^, *M*_w_ = 10,100 g mol^−1^, PDI = 1.9 and Dp = 6. ^1^H NMR (toluene fraction) (C_2_D_2_Cl_4_, δ): 8.08 (d, 2H, *J* = 9.0 Hz), 7.52–7.16 (bm, 4H), 3.85–3.66 (bm, 2H), 2.01–1.83 (bm, 4H), 1.81–1.68 (bm, 1H), 1.61–1.48 (bm, 3H), 1.47–1.22 (bm, 12H), 1.20–0.92 (bm, 12H), 0.90–0.82 (bm, 9H), 0.81–0.72 (bm, 6H), 0.69 (t, 6H, *J* = 7.5 Hz). FT-IR (cm^−1^): 3127, 3074, 2953, 2921, 2850, 1751, 1698, 1513, 1432, 1361, 1169, 1063.

Synthesis of poly[4,4-dioctyl-2,6-cyclopenta[2,1-*b*:3,4-*b*′]dithiophene-*alt*-5,5-(4′,7′-bis(2-thienyl)-2′,1′,3′-benzothiadiazole-5,6-*N*-(3,7-dimethyloctyl)-dicarboxylic imide)] (PCPDTDTBTDI-8, DMO): M4 (109.9 mg, 0.196 mmol), M1 (100 mg, 0.196 mmol), Pd_2_(dba)_3_.CHCl_3_ (6.2 mg, 0.005 mmol), P(*o*-MeOPh)_3_ (8.4 mg, 0.023 mmol), PivOH (20 mg, 0.196 mmol), and Cs_2_CO_3_ (191.7 mg, 0.588 mmol) were wadded to a top sealing tube and degassed under argon. Dry toluene: DMF (2 mL: 0.2 mL) was added and the system was degassed again and heated at 115 °C for 17 h. The reaction contents were cooled to RT, the polymer was dissolved in CHCl_3_ (300 mL), and an NH_4_OH solution (50 mL, 35% in H_2_O) was added and stirred overnight. The organic phase was separated and washed with deionized H_2_O. The organic phase was concentrated (to around 50 mL) and put into methanol (300 mL). The mixture was stirred overnight and filtered. The polymer was purified by Soxhlet extraction with methanol (300 mL), acetone (300 mL), and hexane (300 mL), and finally toluene (300 mL). The toluene fraction was concentrated (to around 50 mL) and put into methanol (300 mL). The solution was stirred overnight, and the pure polymer was recovered by filtration to yield PCPDTDTBTDI-8, DMO as dark green powders (170 mg, 0.19 mmol, 95% yield) [41]. GPC: toluene fraction, *M*_n_ = 7800 g mol^−1^, *M*_w_ = 18,100 g mol^−1^, PDI = 2.3 and Dp = 9. ^1^H NMR (toluene fraction) (C_2_D_2_Cl_4_, δ): 8.04 (bm, 2H), 7.35–7.06 (bm, 4H), 3.83–3.69 (bm, 2H), 1.98–1.84 (bm, 4H), 1.80–1.67 (bm, 1H), 1.62–1.47 (bm, 3H), 1.45–1.13 (bm, 23H), 1.12–1.03 (bm, 3H), 1.01–0.93 (bm, 3H), 0.88–0.77 (bm, 16H). FT-IR (cm^−1^): 3131, 3067, 2921, 2847, 1744, 1694, 1503, 1432, 1396, 1173, 1105, 1066. Elemental analysis (%) calculated for C_51_H_61_N_3_O_2_S_5_: C, 67.44; H, 6.77; N, 4.63; S, 17.65. Found: C, 61.10; H, 7.02; N, 3.98; S, 15.38.

PCPDTDTBTDI-8, DMO was prepared for the second time by the same procedure except the polymerization was left for 72 h. Toluene fraction (110 mg, 0.12 mmol, 62% yield) and chloroform fraction (42 mg, 0.05 mmol, 24% yield), with total yield of 86% [41]. GPC: toluene fraction, *M*_n_ = 10,000 g mol^−1^, *M*_w_ = 30,900 g mol^−1^, PDI = 3.0 and Dp = 11; chloroform fraction, *M*_n_ = 17,400 g mol^−1^, *M*_w_ = 61,400 g mol^−1^, PDI = 3.5 and Dp = 19. 

Synthesis of poly[4,4-dioctyl-2,6-cyclopenta[2,1-*b*:3,4-*b*′]dithiophene-*alt*-5,5-(4′,7′-bis(2-thienyl)-2′,1′,3′-benzothiadiazole-5,6-*N*-octyl-dicarboxylic imide)] (PCPDTDTBTDI-8, 8): PCPDTDTBTDI-8, 8 was prepared following the same procedure for synthesis of PCPDTDTBTDI-8, DMO. M4 (101.2 mg, 0.180 mmol) and M2 (86.9 mg, 0.180 mmol) were copolymerized in the presence of Pd_2_(dba)_3_.CHCl_3_ (5.5 mg, 0.0053 mmol), P(*o*-MeOPh)_3_ (7.6 mg, 0.0216 mmol), PivOH (18.4 mg, 0.180 mmol), Cs_2_CO_3_ (176.4 mg, 0.54 mmol), and anhydrous toluene: DMF (2 mL: 0.2 mL) for 51 h to afford PCPDTDTBTDI-8, 8 as dark green powders (114 mg, 0.13 mmol, 72% yield) [41]. GPC: toluene fraction, *M*_n_ = 4900 g mol^−1^, *M*_w_ = 20,800 g mol^−1^, PDI = 4.2 and Dp = 6. ^1^H NMR (toluene fraction) (C_2_D_2_Cl_4_, δ): 8.07 (bm, 2H), 7.35–7.18 (bm, 4H), 3.83–3.66 (bm, 2H), 1.98–1.83 (bm, 4H), 1.81–1.66 (bm, 2H), 1.48–1.04 (bm, 30H), 0.90–0.78 (bm, 13H). FT-IR (cm^−1^): 3127, 3067, 2918, 2850, 1747, 1698, 1510, 1432, 1393, 1166, 1010. Elemental analysis (%) calculated for C_49_H_57_N_3_O_2_S_5_: C, 66.86; H, 6.53; N, 4.77; S, 18.21. Found: C, 67.23; H, 6.60; N, 4.41; S, 17.45.

PCPDTDTBTDI-8, 8 was prepared for the second time by the same procedure except the polymerization was left for 96 h. Toluene fraction (120 mg, 0.14 mmol, 76% yield) [41]. GPC: toluene fraction, *M*_n_ = 9100 g mol^−1^, *M*_w_ = 18,300 g mol^−1^, PDI = 2.0 and Dp = 10.

Synthesis of poly[4,4-bis(2-ethylhexyl)-2,6-cyclopenta[2,1-*b*:3,4-*b*′]dithiophene-*alt*-5,5-(4′,7′-bis(2-thienyl)-2′,1′,3′-benzothiadiazole-5,6-*N*-octyl-dicarboxylic imide)] (PCPDTDTBTDI-EH, 8): PCPDTDTBTDI-EH, 8 was prepared following the same procedure for synthesis of PCPDTDTBTDI-8, DMO. M5 (104 mg, 0.185 mmol) and M2 (89.3 mg, 0.185 mmol) were copolymerized in the presence of Pd_2_(dba)_3_.CHCl_3_ (5.8 mg, 0.0056 mmol), P(*o*-MeOPh)_3_ (7.8 mg, 0.022 mmol), PivOH (18 mg, 0.185 mmol), Cs_2_CO_3_ (180 mg, 0.55 mmol), and anhydrous toluene: DMF (2 mL: 0.2 mL) for 96 h to afford PCPDTDTBTDI-EH, 8 as dark green powders (118 mg, 0.13 mmol, 72% yield) [41]. GPC: toluene fraction, *M*_n_ = 15,900 g mol^−1^, *M*_w_ = 29,700 g mol^−1^, PDI = 1.8 and Dp = 18. ^1^H NMR (toluene fraction) (C_2_D_2_Cl_4_, δ): 8.15–8.02 (bm, 2H), 7.35–7.20 (bm, 4H), 3.83–3.66 (bm, 2H), 2.07–1.89 (bm, 4H), 1.81–1.70 (bm, 2H), 1.48–1.22 (bm, 18H), 1.17–0.97 (bm, 10H), 0.94–0.84 (bm, 3H), 0.83–0.76 (bm, 6H), 0.75–0.69 (bm, 6H). FT-IR (cm^−1^): 3131, 3070, 2921, 2854, 1751, 1698, 1527, 1432, 1396, 1166, 1098. Elemental analysis (%) calculated for C_49_H_57_N_3_O_2_S_5_: C, 66.86; H, 6.53; N, 4.77; S, 18.21. Found: C, 67.09; H, 6.41; N, 4.57; S, 17.65.

## 3. Results and Discussion

### 3.1. Monomers and Polymers Synthesis

**Scheme 1:**
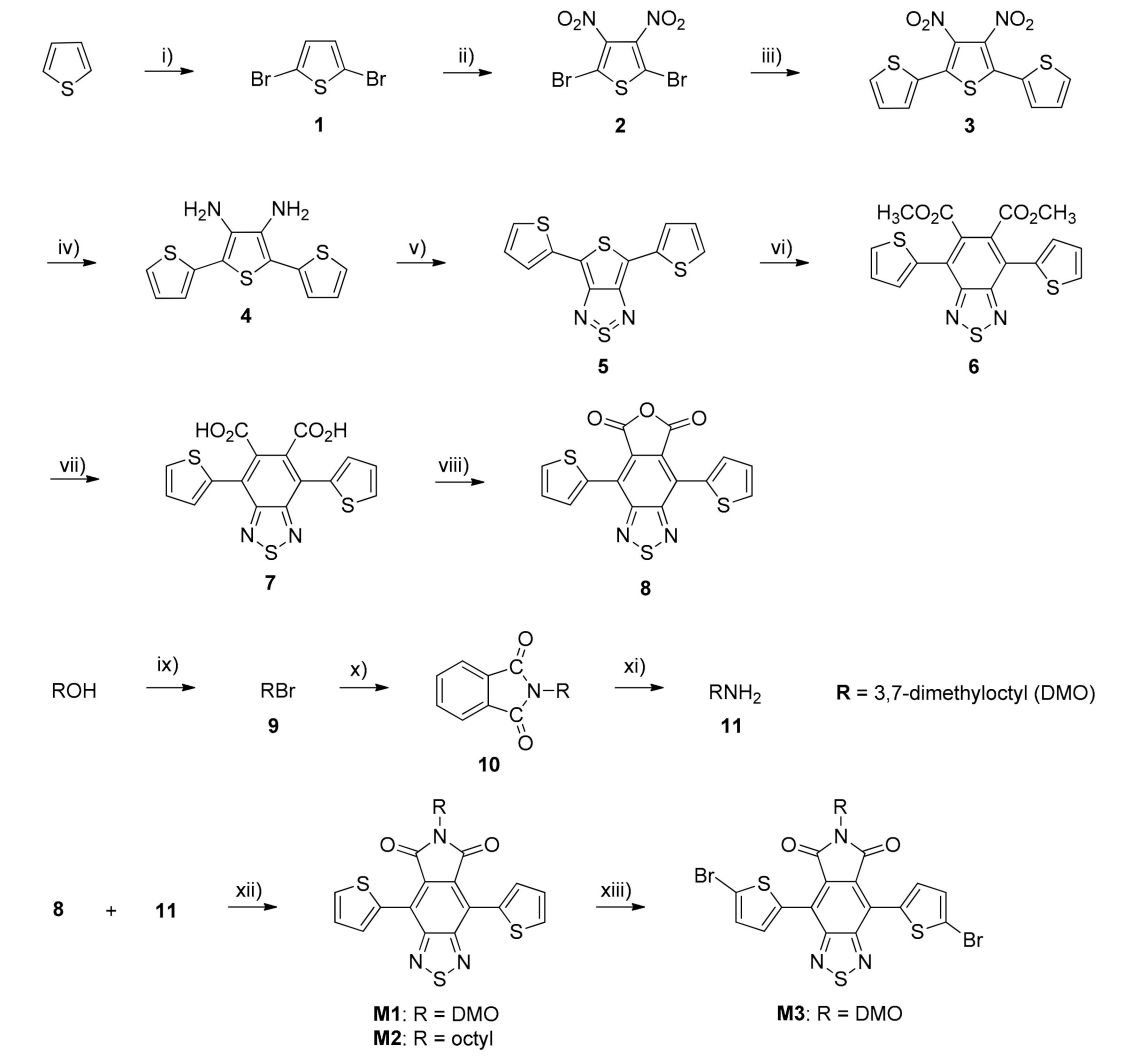
Synthetic steps of the M1, M2, and M3. Reagents and conditions: (i) NBS, DMF, −15 °C, RT, overnight; (ii) fuming H_2_SO_4_ (20% free SO_3_), conc. H_2_SO_4_, conc. HNO_3_, 20 °C, 20–30 °C, 3 h; (iii) 2-(tributylstannyl)thiophene, anhydrous toluene, PdCl_2_(PPh_3_)_2_, 115 °C, 24 h; (iv) anhydrous SnCl_2_, HCl (35%), ethanol, 30 °C, 24 h, NaOH (25%); (v) PhNSO, TMSCl, anhydrous pyridine, 3 h, RT, HCl (1.0 N); (vi) dimethyl acetylenedicarboxylate, anhydrous xylene, reflux 24 h; (vii) aqueous NaOH, ethanol, reflux 24 h, HCl (35%); (viii) anhydrous Ac_2_O, anhydrous xylene, 130 °C, 6 h; (ix) DCM, PPh_3_, NBS, RT, 90 min; (x) potassium phthalimide, anhydrous DMF, 90 °C, 17 h, KOH; (xi) hydrazine hydrate (51%), methanol, reflux, HCl (5.0 M), reflux, 1 h; (xii) HOAc (100%), 100 °C overnight, Ac_2_O, 100 °C, 6 h; (xiii) NBS, THF, RT, overnight.

The synthetic methods for the preparation of benzothiadiazole dicarboxylic imide (BTDI) monomers (M1, M2, and M3) are outlined in Scheme 1. M1, M2, and M3 were synthesized through several steps starting from commercially available thiophene. For the synthesis of 2,5-dibromothiophene (1), thiophene was selectively brominated at 2,5-positions using two equivalents of *N*-bromosuccinimide (NBS) in DMF in the dark to give 1 as a yellow oily product in a high yield [22]. Then, 1 was nitrated with concentrated nitric acid/sulphuric acid and fuming sulphuric acid to give 2,5-dibromo-3,4-dinitrothiophene (2) [23,42,43]. The compound 2 was then reacted with 2-(tributylstannyl)thiophene in the presence of PdCl_2_(PPh_3_)_2_ as a catalyst in anhydrous toluene at 115 °C to give 3′,4′-dinitro-2,2′:5′,2″-terthiophene (3). The resulting compound was obtained as orange crystals in an excellent yield of 90% [24]. Further, 3′,4′-diamino-2,2′:5,2″-terthiophene (4) was obtained by a reduction reaction of 3 using excess anhydrous tin(II) chloride (SnCl_2_). Then, 4 was achieved as a brown solid in an excellent yield of 97% [25]. The resulting substance (4) was then reacted with *N*-thionyl aniline (PhNSO) and trimethylsilyl chloride (TMSCl) in anhydrous pyridine to afford 4,6-bis(2-thienyl)-thieno[3,4-*c*][1,2,5]-thiadiazole (5) as blue crystals in an excellent yield of 93% [26]. The compound 4,7-di(thien-2-yl)-2,1,3-benzothiadiazole-5,6-dimethyl ester (6) was obtained by the Diels-Alder reaction between 5 and dimethyl acetylenedicarboxylate in anhydrous xylene at reflux. It was obtained in an excellent yield of 94% as yellow crystals [27]. Next, the material 6 was hydrolysed under basic conditions in ethanol under reflux followed by acidification to yield 4,7-di(thien-2-yl)-2,1,3-benzothiadiazole-5,6-dicarboxylic acid (7) as a yellow solid in a yield of 85% [28]. The compound 4,7-di(thien-2-yl)-2,1,3-benzothiadiazole-5,6-dicarboxylic anhydride (8) was synthesized by intramolecular ring closure of 7, in the presence of acetic anhydride and anhydrous xylene at 130 °C, to afford 8 as a red solid in an excellent yield of 97% [29]. Moreover, 3,7-dimethyloctyl bromide (9) was synthesized from the reaction of commercially available 3,7-dimethyl-1-octanol with triphenylphosphine (Ph_3_P)/NBS in dichloromethane to yield 9 as a colourless oil in 73% yield [30]. Then, 9 was reacted with potassium phthalimide in anhydrous DMF to give *N*-(3,7-dimethyloctyl)phthalimide (10) as a colourless oil in an excellent yield of 91% [31]. 3,7-dimethyl-1-octanamine (11) was obtained by Gabriel synthesis from the reaction of 10 with hydrazine hydrate (NH_2_NH_2_) in methanol as brown oil in 86% yield [44]. The compounds 4,7-di(thien-2-yl)-2,1,3-benzothiadiazole-5,6-*N*-(3,7-dimethyloctyl)dicarboxylic imide (M1) and 4,7-di(thien-2-yl)-2,1,3-benzothiadiazole-5,6-*N*-octyl-dicarboxylic imide (M2) were synthesized by the reaction of 8 with 11 and 1-octanamine in the presence of acetic acid and acetic anhydride to yield imide functionalized monomers (M1 and M2) as orange solids in 84% and 93% yield, respectively [29]. Lastly, the monomer 4,7-di(5-bromo-thien-2-yl)-2,1,3-benzothiadiazole-5,6-*N*-(3,7-dimethyloctyl)dicarboxylic imide (M3) was prepared by the bromination of M1 at 5,5′-positions using NBS in THF and to yield M3 as red solids in excellent yields of 98% [28].

**Scheme 2:**
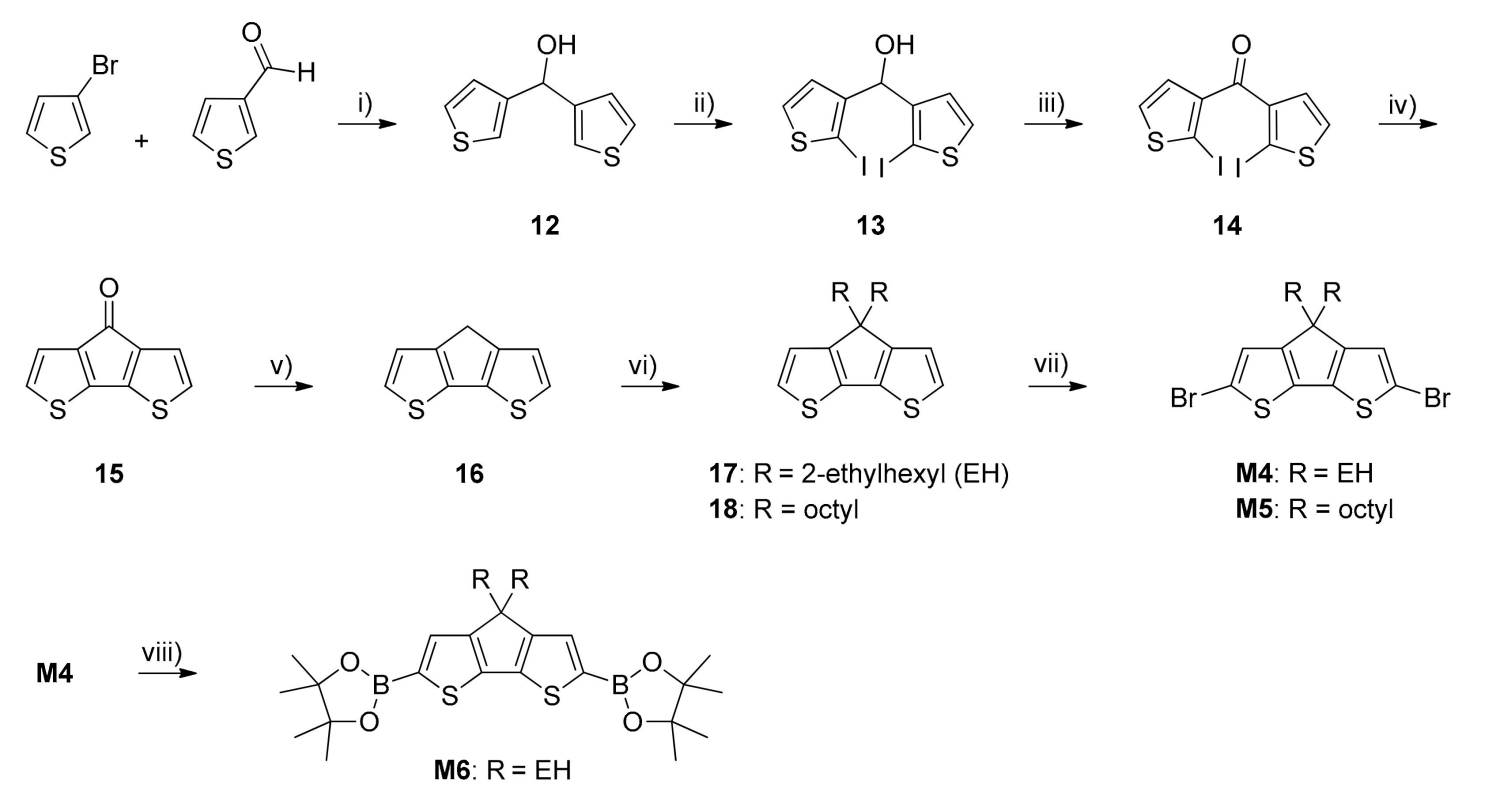
The synthetic steps of the M4, M5, and M6. **Reagents and conditions:** (i) *n*-BuLi, anhydrous Et_2_O, −78 °C; (ii) *n*-BuLi, anhydrous Et_2_O, −78 °C, I_2_; (iii) PCC, CH_2_Cl_2_, RT, 24 h; (iv) Cu, anhydrous DMF, reflux, 150 °C, 22 h; (v) triethylene glycol, KOH, hydrazine hydrate, 180 °C, 17 h; (vi) KOH, KI, anhydrous DMSO, R-Br, RT, 24 h; (vii) NBS, DMF, RT, 24 h; and (viii) bis(pinacolato)diboron, PdCl_2_(dppf), KOAc, anhydrous DMF, 80 °C, 48 h.

The synthetic methods for the preparation of cyclopentadithiophene (CPDT) monomers (M4, M5, and M6) were synthesized starting from commercially available 3-bromothiophene and thiophene-3-carboxaldehyde as shown in Scheme 2. 

For the synthesis of bis(3-thienyl)methanol (12), 3-Bromothiophene was lithiated selectively at the 3-position at −78 °C and subsequently treated with thiophene-3-carboxaldehyde to obtain 12 as a yellow oil in 85% yield [33]. Bis(2-iodo-3-thienyl)methanol (13) was synthesized from 12. In this reaction, 12 was lithiated selectively at 2,2′-positions and subsequently reacted with iodine to yield 13 as cream-coloured crystals in 79% yield [34]. The synthesis of bis(2-iodo-3-thienyl)ketone (14) was carried out by oxidizing 13 using pyridinium chlorochromate (PCC) as an oxidizing agent in dichloromethane at room temperature to obtain 14 as yellow crystals in 97% yield [45]. *4H*-cyclopenta[2,1-*b*:3,4-*b*′]dithiophen-4-one (15) was prepared by the Ullmann coupling reaction between 14 with copper powder in anhydrous DMF and it was obtained as purple crystals in 98% yield [35]. The compound 15 was then reduced to *4H*-cyclopenta[2,1-*b*:3,4-*b*′]dithiophene (16) using hydrazine hydrate (NH_2_NH_2_) in triethylene glycol under basic conditions, and it was obtained as white crystals in 80% yield by Wolff–Kishner reduction [36]. Afterward, 16 was alkylated under basic conditions with 2-ethylhexyl bromide and 1-bromooctane using small catalytic amount of potassium iodide in anhydrous DMSO and gave 4,4-bis(2-ethylhexyl)cyclopenta[2,1-*b*:3,4-*b*′]dithiophene (17) and 4,4-dioctylcyclopenta[2,1-*b*:3,4-*b*′]dithiophene (18) as yellow oil in 89% and 91% yield, respectively [37].

Next, 17 and 18 were brominated regioselectively at the 2,6-positions with two equivalents of NBS in DMF and yielded 4,4-bis(2-ethylhexyl)-2,6-dibromocyclopenta[2,1-*b*:3,4-*b*′]dithiophene (M4) and 4,4-dioctyl-2,6-dibromocyclopenta[2,1-*b*:3,4-*b*′]dithiophene (M5) as yellow oil in 88% and 87% yield, respectively [38]. Finally, M4 was reacted with excess of bis(pinacolato)diboron, potassium acetate as a base, and PdCl_2_(dppf) as a catalyst in anhydrous DMF to yield 4,4-bis(2-ethylhexyl)-2,6-bis(4,4,5,5-tetramethyl-1,3,2-dioxaboralan-2-yl)cyclopenta[2,1-*b*:3,4-*b*′]dithiophene (M6) as a red sticky oil in 57% yield [39]. It was sticky oily material and then was used for the Suzuki polymerization without further purification. 

### 3.2. Polymers Synthesis

**Scheme 3:**
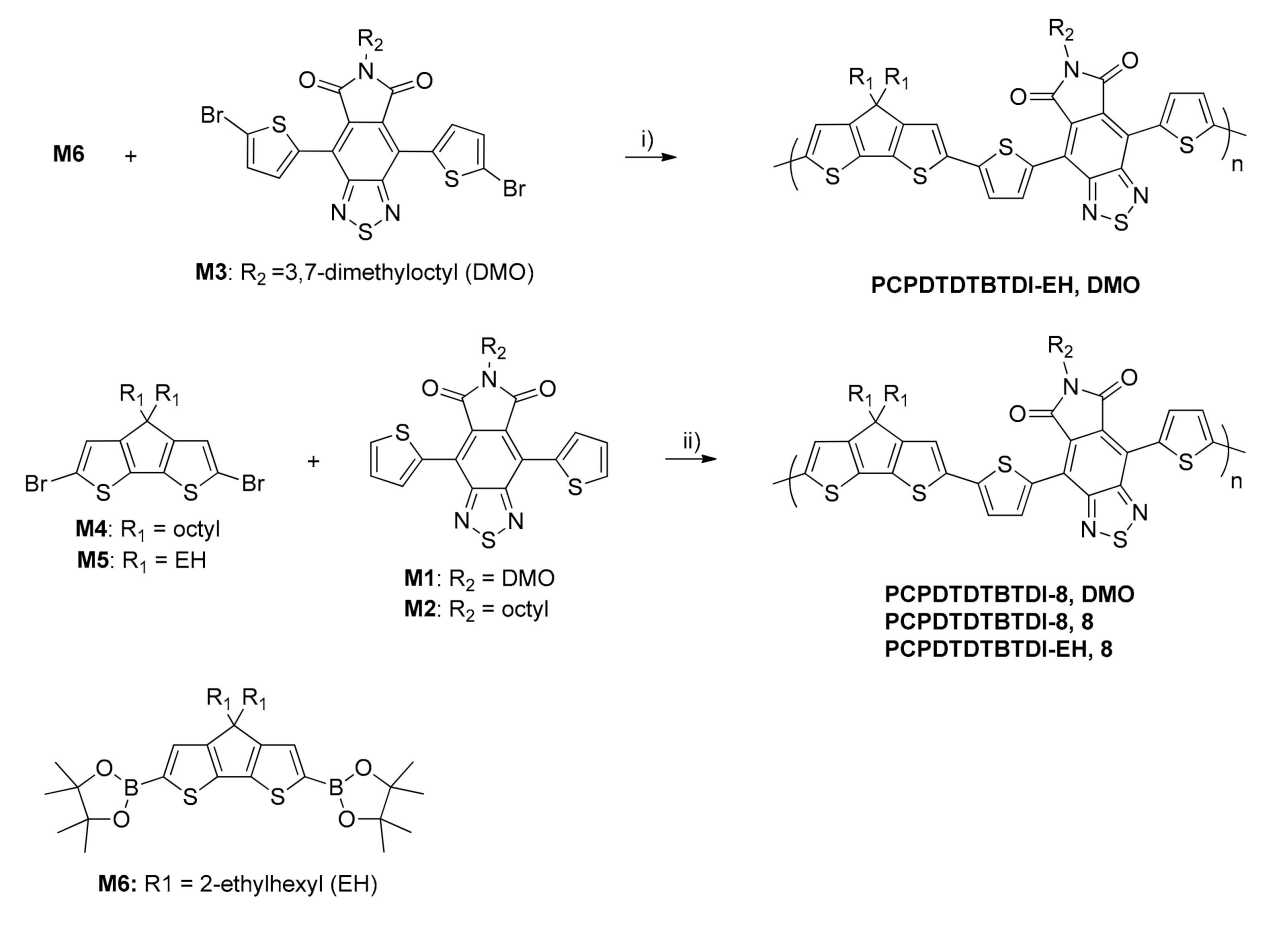
The synthesis of PCPDTDTBTDI-EH, DMO, PCPDTDTBTDI-8, DMO, PCPDTDTBTDI-8, 8, and PCPDTDTBTDI-EH, 8. **Reagents and conditions:** (i) anhydrous THF, NaHCO_3_, Pd(OAc)_2_, P(*o*-tol)_3_, 90 °C, 48 h and (ii) Pd_2_(dba)_3_.CHCl_3_, P(*o*-MeOPh)_3_, PivOH, Cs_2_CO_3_, anhydrous toluene: anhydrous DMF (10:1, *v*/*v*), 115 °C, 17–96 h.

In this work, the preparations of four low band gap copolymers either through Suzuki or direct arylation polymerizations are discussed. PCPDTDTBTDI-EH, DMO was synthesized via Suzuki polymerization between diboronic ester of CPDT monomer (M6) and dibrominated BTDI monomer (M3) (Scheme 3). A direct arylation polymerization as a new synthetic method was used to prepare the other three copolymers based on CPDT units. One of the advantages of the direct arylation polymerization is that it requires fewer synthetic steps compared to Suzuki and Stille polymerizations [41]. It eliminates the need to prepare boronic ester and stannylated monomeric compounds, which are sometimes challenging to purify from their by-products as in the case for M6. It also avoids the use of toxic compounds especially tin compounds [41]. PCPDTDTBTDI-8, DMO, PCPDTDTBTDI-8, 8 and PCPDTDTBTDI-EH, 8 were synthesized successfully through direct arylation polymerization using Pd_2_(dba)_3_.CHCl_3_/P(*o*-MeOPh)_3_ catalyst, caesium carbonate base, and pivalic acid in anhydrous toluene: DMF as a cosolvent. M4 was copolymerized with both M1 and M2 to form PCPDTDTBTDI-8, DMO and PCPDTDTBTDI-8, 8, respectively. PCPDTDTBTDI-EH, 8 was obtained by copolymerizing M5 with M2. All polymerizations were left running between 17 and 96 h with large amounts of dark green precipitates forming as the reactions proceeded. The polymers were then dissolved in chloroform and an ammonia solution was added, and then, the mixture was stirred overnight to remove the Pd metal catalyst residues by forming Pd(NH_3_)_4_(OH)_2_ soluble complexes. The polymers were obtained by precipitation from methanol followed by filtration. The polymers were purified via Soxhlet extraction with methanol, acetone, hexane, toluene, and finally chloroform. The small molecules, oligomers, and impurities were removed in the methanol, acetone, and hexane fractions. The toluene and chloroform fractions of the polymers were subsequently collected and concentrated in vacuo and reprecipitated in methanol followed by filtration to yield the purified polymers. The structures of the PCPDTDTBTDI-EH, DMO, PCPDTDTBTDI-8, DMO, PCPDTDTBTDI-8, 8, and PCPDTDTBTDI-EH, 8 were confirmed by the ^1^H NMR spectroscopy, FT-IR spectroscopy, and elemental analysis. The ^1^H NMR spectra of the polymers are available in the Supplementary Materials Information.

### 3.3. Molecular Weights and Yields

Molecular weights of the polymers were measured by GPC in chloroform solution at 40 °C relative to polystyrene standards (Table 1). Although, the polymerization of PCPDTDTBTDI-EH, DMO was performed for 48 h, it was synthesized in very low yield (˂10%) and with a low number average molecular weight (*M*_n_ ~ 5000 g mol^−1^). There are several factors that would lead to the low *M*_n_ value and low yield of this polymer. One of the main reasons is probably due to severe steric hindrance between two branched alkyl chains (2-ethylhexyl and 3,7-dimethyloctyl) on CPDT and BTDI repeat units, respectively. The second reason is that the bis-boronic ester monomer (M6) contains some impurities such as unreacted staring material and monoboronic ester, since M6 was used for polymerization without further purification. Since the monomer was unstable, it was difficult to purify by column chromatography. Finally, the polymer contains too much solubilizing side chains. It is well known that both steric hindrance and impurities disrupt the effective conjugation length (ECL) and lead to low molecular weight polymers. PCPDTDTBTDI-8, DMO and PCPDTDTBTDI-8, 8 were synthesized twice under the same experimental conditions but with different reaction times. Substituting 3,7-dimethyloctyl chains in PCPDTDTBTDI-8, DMO for *n*-octyl chains in PCPDTDTBTDI-8, 8 on the BTDI units led to lower *M*_n_ values for the toluene fractions of the polymers in the first polymerization. However, in the second polymerization, by extending the reaction time, the *M*_n_ value of the former polymer increased slightly, while *M*_n_ value of the latter polymer increased significantly for the toluene fractions. In addition, PCPDTDTBTDI-8, DMO afforded another fraction in chloroform of a higher *M*_n_ value. The higher molecular weight polymers could be obtained by prolonging the polymerization times as well as by substituting *n*-octyl chains to 3,7-dimethyloctyl chains on the BTDI building blocks. Furthermore, replacing *n*-octyl chains in PCPDTDTBTDI-8, 8 by 2-ethylhexyl chains in PCPDTDTBTDI-EH, 8 on the CPDT moieties results in a polymer with the higher *M*_n_ value for toluene fractions. Moreover, PCPDTDTBTDI-EH, 8 has the highest *M*_n_ value for toluene fractions among all polymers prepared. This could be attributed to the effect of the branched chains in both PCPDTDTBTDI-8, DMO and PCPDTDTBTDI-EH, 8, which provide greater solubilities and higher molecular weight fractions. The yields of the direct arylation polymerization are high between 72% and 95%, and PCPDTDTBTDI-8, DMO has the highest yield. 

### 3.4. Optical Properties

**Figure 1:**
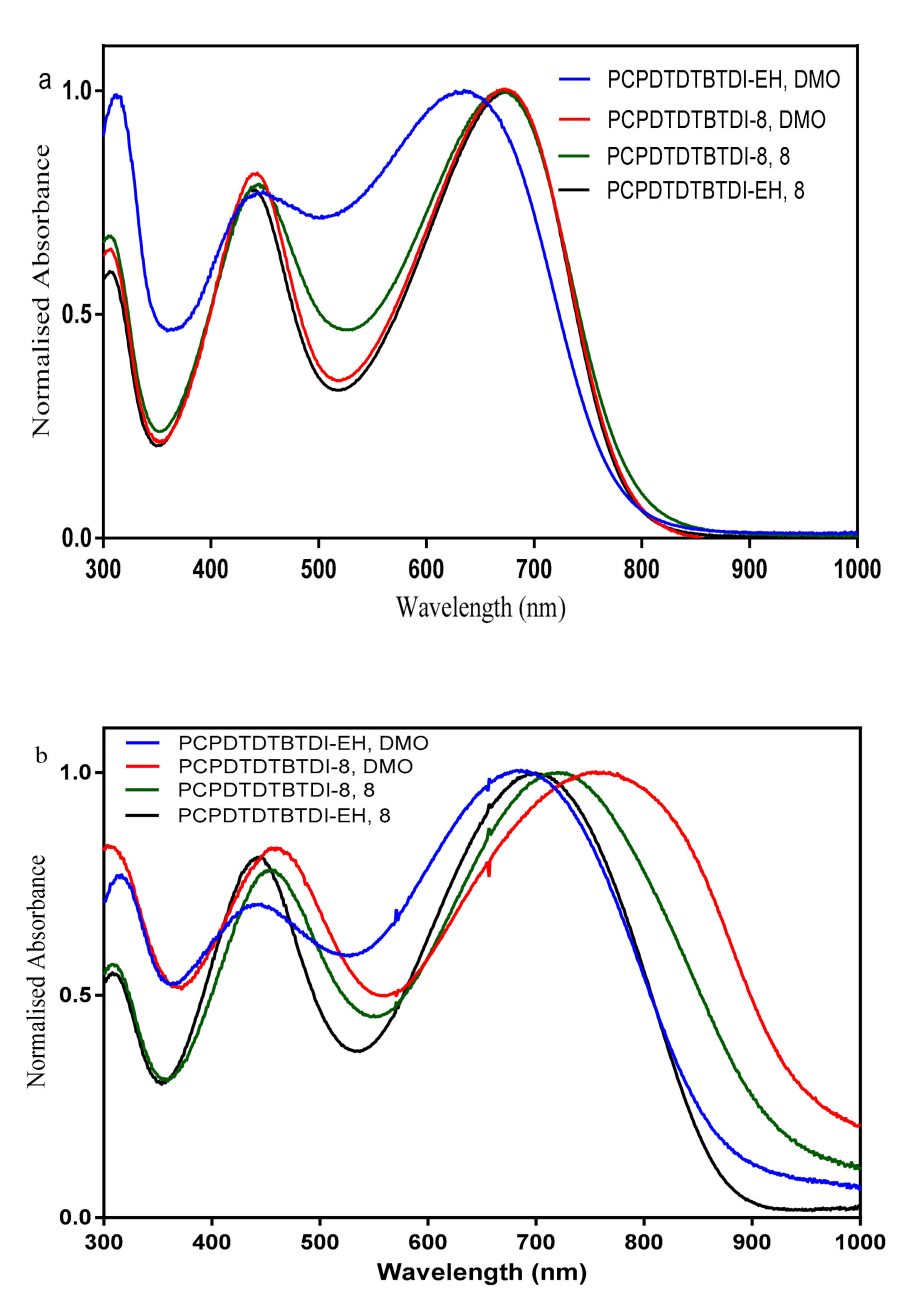
Normalized UV-VIS absorption spectra of PCPDTDTBTDI-EH, DMO, PCPDTDTBTDI-8, DMO, PCPDTDTBTDI-8, 8, and PCPDTDTBTDI-EH, 8 in (**a**) chloroform solutions and (**b**) thin films.

UV-visible spectrophotometer is an efficient technique used to study the UV-VIS absorption spectra of various types of conductive polymers, polymer electrolytes, and composites [46,47,48,49,50]. The UV-VIS absorption spectra of all polymers were investigated in chloroform solutions and in thin films (Figure 1 and Table 2). In solutions, PCPDTDTBTDI-8, DMO, PCPDTDTBTDI-8, 8, and PCPDTDTBTDI-EH, 8 display comparable absorption maxima, which are red-shifted around 40 nm relative to those of PCPDTDTBTDI-EH, DMO analogue probably as a result of the low molecular weight of the latter polymer. In thin films, the absorption spectra of the polymers show quite strong bathochromic shift absorption maxima between 29 and 86 nm relative to their absorption in solutions. This could be explained by stronger intermolecular π–π interaction and more planar structures in the solid state. The E_g_ of the polymers is estimated from the absorption onsets in thin films. PCPDTDTBTDI-8, DMO and PCPDTDTBTDI-8, 8 have comparable E_g_ (*ca.* 1.3 eV), which is around 0.1 eV lower than those of PCPDTDTBTDI-EH, DMO and PCPDTDTBTDI-EH, 8 analogues. The results indicate that substituting 2-ethylhexyl chains by *n*-octyl chains on CPDT units would lead to lower E_g_ of the polymers, while changing 3,7-dimethyloctyl chains by *n*-octyl chains on BTDI moieties has a minimal effect on the E_g_ of the polymers. These polymers are good candidates as donor materials fabricated with fullerene derivatives as top BHJ cell in tandem solar cells due to their low optical band gaps [51]. 

The E_g_ values of PCPDTDTBTDI-EH, DMO and PCPDTDTBTDI-EH, 8 are comparable to PCPDTBT analogue [4]. However, PCPDTDTBTDI-8, DMO and PCPDTDTBTDI-8, 8 have lower E_g_ values around 0.1 eV relative to PCPDTBT counterpart. The E_g_ of the polymers is significantly lower than those of thienopyrroledione-, bithiazole-, and tetrazine-based polymers because the BTDI unit is stronger acceptor than those units [14,15,16,17,18,19]. The optical band gaps of PCPDTDTBTDI-EH, DMO, PCPDTDTBTDI-8, DMO, PCPDTDTBTDI-8, 8, and PCPDTDTBTDI-EH, 8 are significantly lower than fluorene-, dibenzosilole-, and carbazole-based D-A copolymers [52,53]. This could be explained by the stronger electron-donating abilities of CPDT units compared to fluorene, dibenzosilole, and carbazole units. As a result, the D-A copolymers based on CPDT units adopt more planar structure with stronger interchain interactions along the polymer backbone.

PCPDTDTBTDI-EH, DMO and PCPDTDTBTDI-EH, 8 have comparable absorption coefficients (ε) and their coefficients are slightly higher than PCPDTDTBTDI-8, 8. PCPDTDTBTDI-8, DMO has the highest absorption coefficient among all polymers prepared. This could be attributed to the PCPDTDTBTDI-8, DMO having the highest absorption maxima of about 759 nm in solid state, and it is red-shifted by more than 80 nm compared to solution among all polymers (Table 2).

### 3.5. Electrochemical Properties 

**Figure 2:**
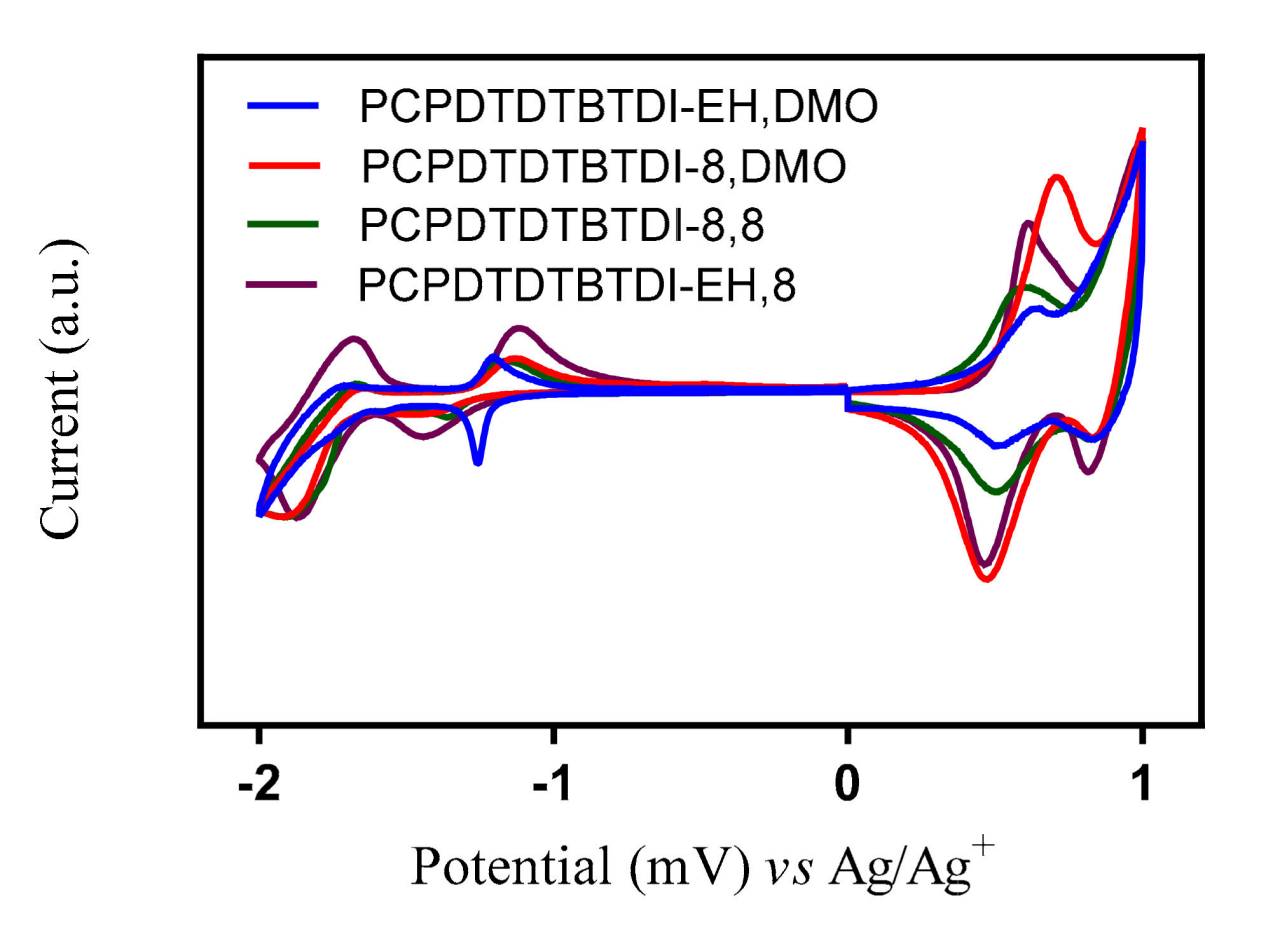
Cyclic voltammograms of PCPDTDTBTDI-EH, DMO, PCPDTDTBTDI-8, DMO, PCPDTDTBTDI-8, 8, and PCPDTDTBTDI-EH, 8 on Pt electrode in Bu_4_NClO_4_/CH_3_CN at 100 mV/s.

Cyclic voltammetry was used to study the electrochemical properties of the polymers. The electrochemical energy storage devices were investigated using cyclic voltammetry [54,55,56,57,58]. The LUMO and HOMO levels of the polymers are calculated from the onsets of reduction and oxidation potentials, respectively (Figure 2 and Table 3). The onsets were determined from cyclic voltammograms on drop-cast polymer films on Pt electrode as working electrode in Bu_4_NClO_4_/CH_3_CN (0.1 M) vs. Ag/Ag^+^ reference electrode. The onset potentials of the first oxidation wave of PCPDTDTBTDI-EH, DMO, PCPDTDTBTDI-8, DMO, PCPDTDTBTDI-8, 8, and PCPDTDTBTDI-EH, 8 appeared at 0.44 0.49, 0.39, and 0.51 V vs. Ag/Ag^+^, corresponding to HOMO energy levels of −5.15, −5.20, −5.10, and −5.22 eV, respectively. The onset potentials of the first reduction wave of PCPDTDTBTDI-EH, DMO, PCPDTDTBTDI-8, DMO, PCPDTDTBTDI-8, 8, and PCPDTDTBTDI-EH, 8 appeared at −3.52, −3.47, −3.44, and −3.54 V corresponding to LUMO energy levels of −3.52, −3.47, −3.44, and −3.54 eV. This corresponds to an electrochemical band gap of 1.63, 1.73, 1.66, and 1.68 eV for PCPDTDTBTDI-EH, DMO, PCPDTDTBTDI-8, DMO, PCPDTDTBTDI-8, 8, and PCPDTDTBTDI-EH, 8, respectively.

The HOMO levels of PCPDTDTBTDI-8, DMO and PCPDTDTBTDI-EH, 8 are −5.20 and −5.22 eV, respectively, which are deeper than PCPDTDTBTDI-EH, DMO and PCPDTDTBTDI-8, 8. The LUMO energy levels of PCPDTDTBTDI-8, DMO and PCPDTDTBTDI-8, 8 are higher than PCPDTDTBTDI-EH, DMO and PCPDTDTBTDI-EH, 8. The HOMO and LUMO levels of the polymers are not significantly affected by different substituents attached to the CPDT and BTDI units. 

The HOMO levels of the polymers are higher around 0.1–0.2 eV, while the LUMO energy levels are almost identical relative to its PCPDTBT analogue [5]. The HOMO levels of the polymers are shallower than the HOMO level of PCPDTTPD (−5.43 eV), while their LUMO energy levels are lower than the LUMO energy level of PCPDTTPD (−3.25 eV) [18].

### 3.6. Thermal Properties 

**Figure 3:**
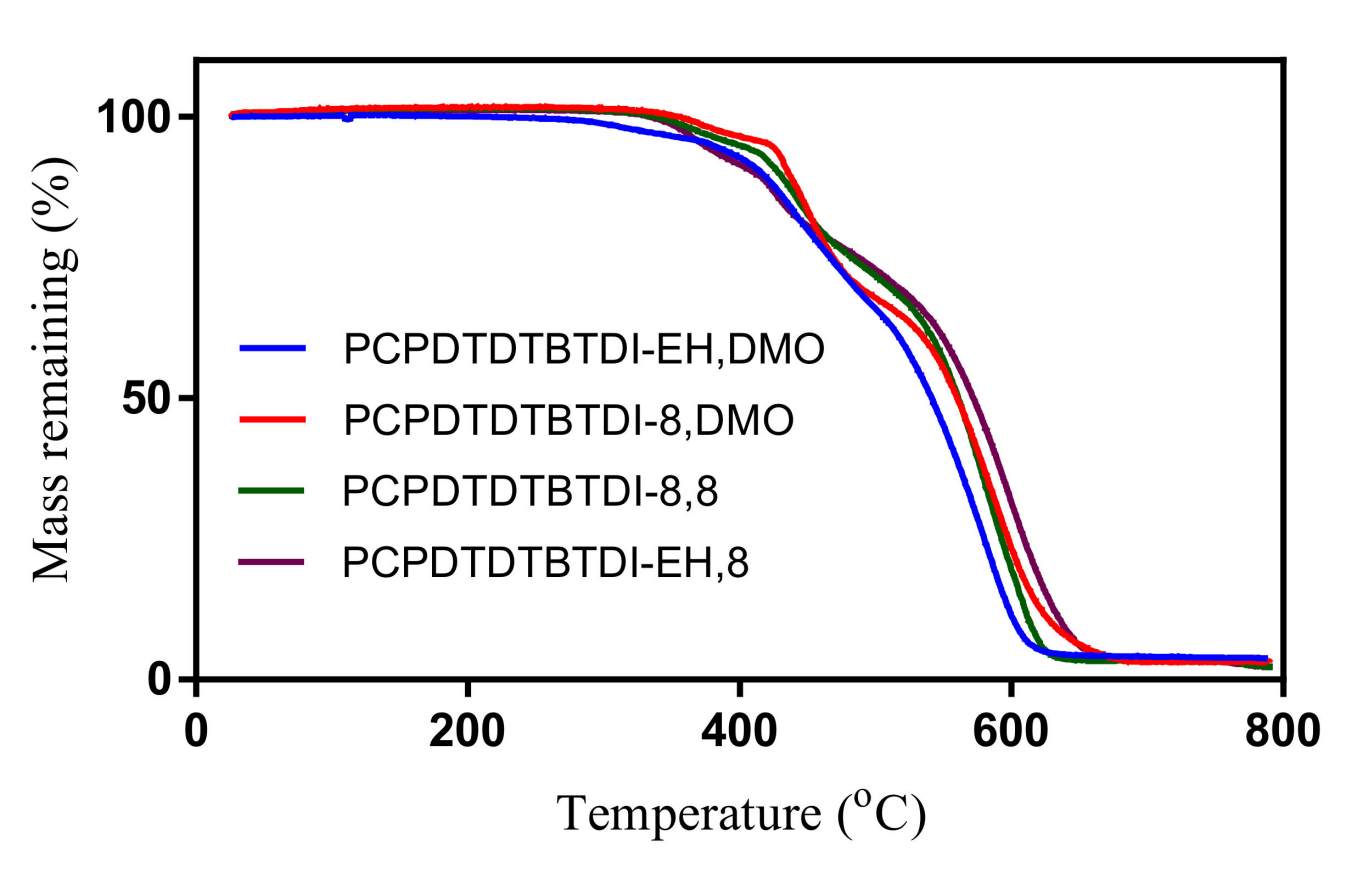
TGA of PCPDTDTBTDI-EH, DMO, PCPDTDTBTDI-8, DMO, PCPDTDTBTDI-8, 8, and PCPDTDTBTDI-EH, 8.

Thermal properties of the polymers were studied by TGA (Figure 3 and Table 3). All polymers show high thermal stability with decomposition temperatures up to 370 °C. It is interesting to note that the thermal stability of the polymers with linear *n*-octyl chains on CPDT repeat units is higher than those polymers with branched 2-ethylhexyl chains. In addition, the thermal stability of the PCPDTDTBTDI-EH DMO and PCPDTDTBTDI-EH, 8 is not affected by the nature of substituents on BTDI units, while changing the 3,7-dimethyloctyl chains in PCPDTDTBTDI-8, DMO to *n*-octyl chains in PCPDTDTBTDI-8, 8 on the acceptor moieties has a negative impact on the thermal stability of the polymers. It was tentatively hypothesized that the polymers with *n*-octyl chains are more planar than those with 2-ethylhexyl chains; therefore, they might need higher temperature to decompose. Moreover, the differences in thermal stability of the polymers are probably due to different molecular weights of the polymers. 

### 3.7. Powder X-ray Diffraction of the Polymers

**Figure 4:**
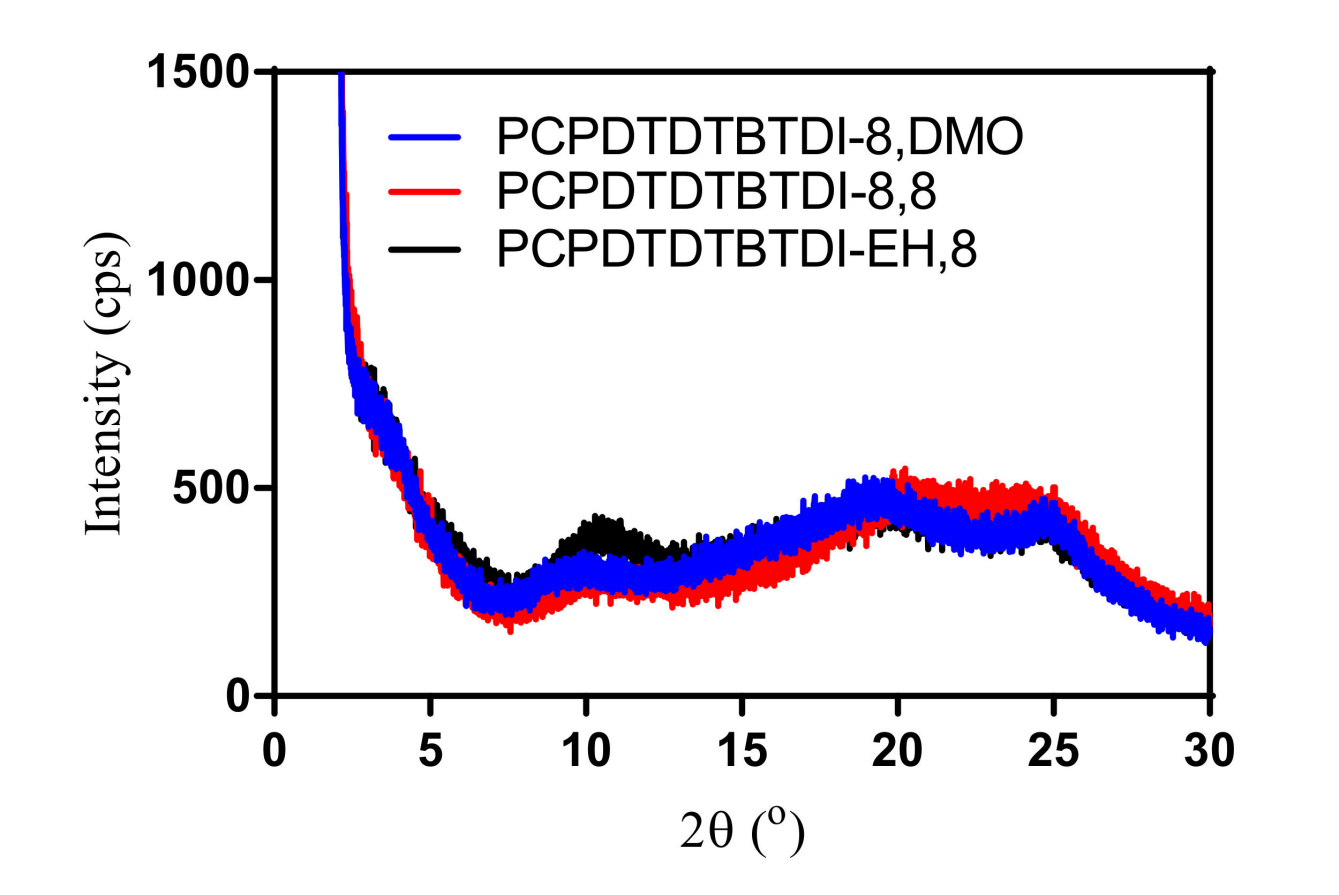
Powder XRD of PCPDTDTBTDI-8, DMO, PCPDTDTBTDI-8, 8, and PCPDTDTBTDI-EH, 8.

X-ray diffraction (XRD) was used to study the amorphous nature of polymer electrolytes and composites [59,60,61,62,63]. The structural properties of the PCPDTDTBTDI-8, DMO, PCPDTDTBTDI-8, 8, and PCPDTDTBTDI-EH, 8 were investigated by powder X-ray diffraction (XRD) in solid state (Figure 4). However, PCPDTDTBTDI-EH, DMO was not studied by powder XRD because the amount obtained from the Suzuki polymerization was not enough to undertake measurements. The XRD results of PCPDTDTBTDI-8, DMO, PCPDTDTBTDI-8, 8, and PCPDTDTBTDI-EH, 8 show diffraction peaks at 24.7, 24.5, and 24.4° corresponding to the π–π stacking distance of 3.60, 3.62, and 3.64 Å, respectively. The results show that all polymers have an amorphous nature. Similarly, PCPDTBT does not show crystallinity by XRD study as reported in previous literature report [64].

## 4. Conclusions

Four novel low band gap alternating copolymers including cyclopentadithiophene (CPDT) flanked by thienyl units as electron donor moieties and benzothiadiazole dicarboxylic imide (BTDI) as electron acceptor units were synthesized via two different palladium catalysed cross coupling polymerizations. PCPDTDTBTDI-EH, DMO was prepared by copolymerizing the diboronic ester of CPDT (M6) with dibrominated BTDI (M3) via Suzuki polymerization. The yield of the polymerization was too low (˂10%), and the polymer had a low *M*_n_ value around 5000 g mol^−1^. To circumvent these issues, direct arylation polymerization as a new alternative preparation method was utilized to prepare the PCPDTDTBTDI-8, DMO, PCPDTDTBTDI-8, 8, and PCPDTDTBTDI-EH, 8. All polymers were synthesized in good yields and they had excellent solubility in common organic solvents. Two distinct side chains (*n*-octyl vs. 2-ethylhexyl) were attached to the CPDT units as well as two different side chains (*n*-octyl vs. 3,7-dimethyloctyl) were anchored to the BTDI units to investigate the effect of these substituents on the solubilities, molecular weights, optical and electrochemical properties, thermal and structural properties of the resulting polymers. Changing 3,7-dimethyloctyl chains on the BTDI units in PCPDTDTBTDI-8, DMO for *n*-octyl chains in PCPDTDTBTDI-8, 8 as well as prolonging the polymerization times had a significant effect on the solubility and also on the *M*_n_ values of the resulting polymers. PCPDTDTBTDI-8, DMO provided a toluene fraction, which had a *M*_n_ value of 10,000 g mol^−1^. In addition to the toluene fraction, another fraction from chloroform was obtained, which had a higher *M*_n_ value of 17,400 g mol^−1^. However, PCPDTDTBTDI-8, 8 was extracted in the toluene fraction, which had the *M*_n_ value of 9100 g mol^−1^. Moreover, substituting *n*-octyl chains in PCPDTDTBTDI-8, 8 for 2-ethylhexyl chains in PCPDTDTBTDI-EH, 8 on the CPDT units yielded the polymer with higher *M*_n_ value of 15,900 g mol^−1^ for the toluene fraction. The polymers with one branched chain on either CPDT or BTDI units can provide greater solubilities and higher molecular weight fractions. In solutions, the polymers, which were synthesized by direct arylation polymerization show comparable absorption maxima and display bathochromic shift around 40 nm relative to PCPDTDTBTDI-EH, DMO. In thin-films, the absorption spectra of the polymers show red-shifted absorption maxima by 29–86 nm relative to their absorption in solutions. The optical band gaps of the polymers with *n*-octyl chains on the CPDT units are about 1.3 eV, which are about 0.1 eV lower than those analogues with 2-ethylhexyl chains. However, substituting 3,7-dimethyloctyl chains by *n*-octyl chains on BTDI units has little influence on the E_g_ of the polymers. The low band gap of these polymers is beneficial to achieve high *J*_sc_ values in BHJ solar cells. These polymers could also be used along with higher band gap conjugated polymers as top cells in tandem solar cells. The HOMO energy levels of the polymers are between −5.10 and −5.22 eV. The LUMO energy levels of the polymers are between −3.44 and −3.54 eV. Both the LUMO and HOMO levels of the polymers are affected around 0.1 eV by attaching different substituents on both CPDT and BTDI repeat units as well as changing the types of polymerizations. All polymers display good thermal stability with *T*_d_ exceeding 370 °C. The polymers based on *n*-octyl chains on CPDT units have higher thermal stability than those polymers with 2-ethylhexyl chains on CPDT units. The powder XRD of the PCPDTDTBTDI-8, DMO, PCPDTDTBTDI-8, 8, and PCPDTDTBTDI-EH, 8 show diffraction peaks at around 24.0° corresponding to the π–π stacking distance of about 3.60 Å. They have an amorphous nature, which could be employed as electrolytes in energy devices.

## Figures and Tables

**Table 1:** The percentage yield, reaction time, weight, and number average molecular weights with polydispersity indexes of PCPDTDTBTDI-EH, DMO, PCPDTDTBTDI-8, DMO, PCPDTDTBTDI-8, 8, and PCPDTDTBTDI-EH, 8.

Polymer	% Yield	Time (h)	Toluene Fraction		
			*M*_n_ (g mol^−1^)	*M*_w_ (g mol^−1^)	PDI
PCPDTDTBTDI-EH, DMO	8	48	5200	10,100	1.9
PCPDTDTBTDI-8, DMO	95	17	7800	18,100	2.3
PCPDTDTBTDI-8, DMO *	86	72	10,000	30,900	3.0
PCPDTDTBTDI-8, 8	72	51	4900	20,800	4.2
PCPDTDTBTDI-8, 8	76	96	9100	18,300	2.0
PCPDTDTBTDI-EH, 8	72	96	15,900	29,700	1.8

* Chloroform fraction: *M*_n_ = 17,400 g mol^−1^, *M*_w_ = 61,400 g mol^−1^, and PDI = 3.5.

**Table 2:** The UV–VIS data and optical band gaps of the polymers.

Polymer	ε (M^−1^ cm^−1^)	Solution	Film		
		λ_max_ (nm)	λ_max_ (nm)	λ_onset_ (nm)	E_g_ (eV)
PCPDTDTBTDI-EH, DMO	28,200	635	686	865	1.43
PCPDTDTBTDI-8, DMO	37,500	673	759	936	1.32
PCPDTDTBTDI-8, 8	27,500	672	724	922	1.34
PCPDTDTBTDI-EH, 8	28,400	675	704	873	1.42

**Table 3:** Thermal and electrochemical properties of the polymers.

Polymer	*T*_d_	E_ox_^0^	HOMO	E_red_^0^	LUMO	E_g (elec)_
	(°C)	(V)	(eV)	(V)	(eV)	(eV)
PCPDTDTBTDI-EH, DMO	378	0.44	−5.15	−1.19	−3.52	1.63
PCPDTDTBTDI-8	419	0.49	−5.20	−1.24	−3.47	1.73
PCPDTDTBTDI-8, 8	402	0.39	−5.10	−1.27	−3.44	1.66
PCPDTDTBTDI-EH, 8	377	0.51	−5.22	−1.17	−3.54	1.68

## Supplementary Materials

The following are available online at https://www.mdpi.com/2073-4360/13/1/63/s1, Figure S1: ^1^H NMR spectrum of PCPDTDTBTDI-EH, DMO in C_2_D_2_Cl_4_ at 100 °C, Figure S2: ^1^H NMR spectrum of PCPDTDTBTDI-8, DMO in C_2_D_2_Cl_4_ at 100 °C. Figure S3: ^1^H NMR spectrum of PCPDTDTBTDI-8, 8 in C_2_D_2_Cl_4_ at 100 °C, Figure S4: ^1^H NMR spectrum of PCPDTDTBTDI-EH, 8 in C_2_D_2_Cl_4_ at 100 °C.
