# Supplementary material for: Fabrication of Alternating Copolymers Based on Cyclopentadithiophene-Benzothiadiazole Dicarboxylic Imide with Reduced Optical Band Gap: Synthesis, Optical, Electrochemical, Thermal, and Structural Properties
Source: Polymers (Basel). 2020 Dec 26;13(1):63. doi: 10.3390/polym13010063 (PMC7795047; doi:10.3390/polym13010063)
Supplement: Supplementary file 1 [file polymers-13-00063-s001.pdf]

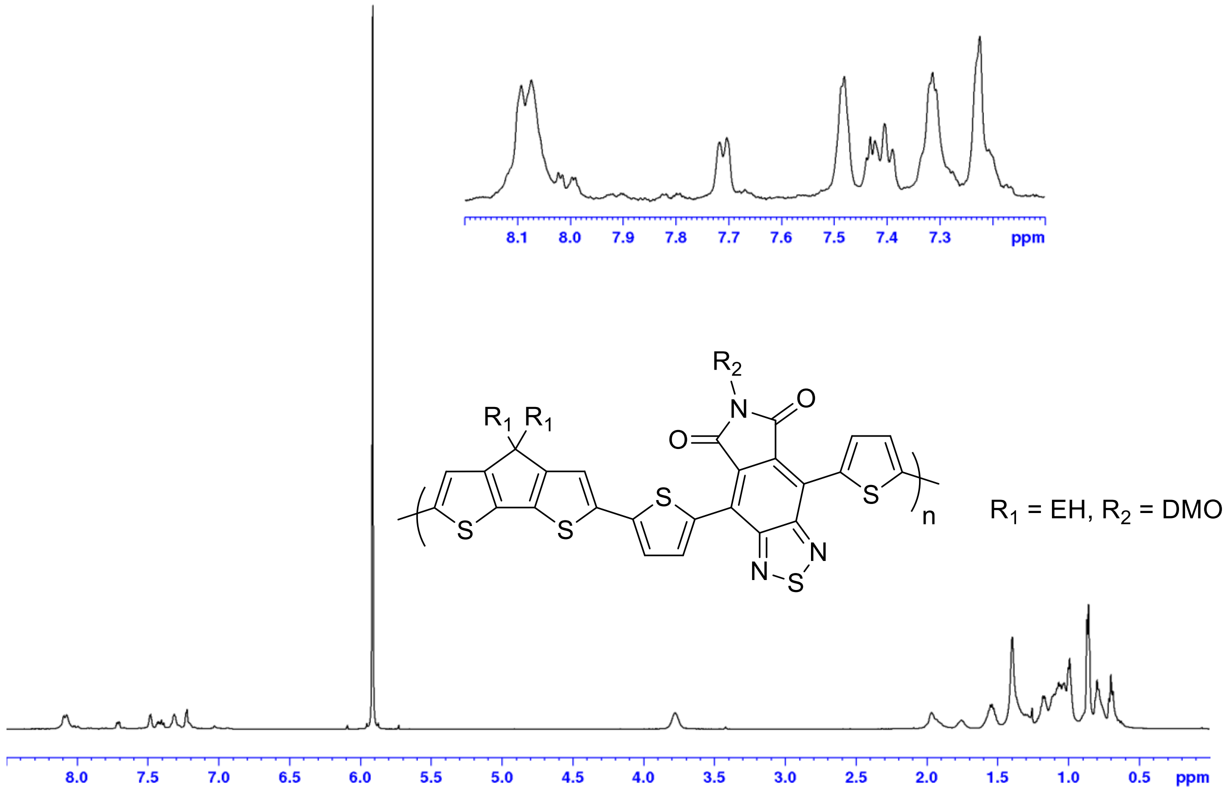

Figure S1. ^{1}H NMR spectrum of PCPDTDTBTDI-EH, DMO in $C_2D_2Cl_4$ at 100 ℃.

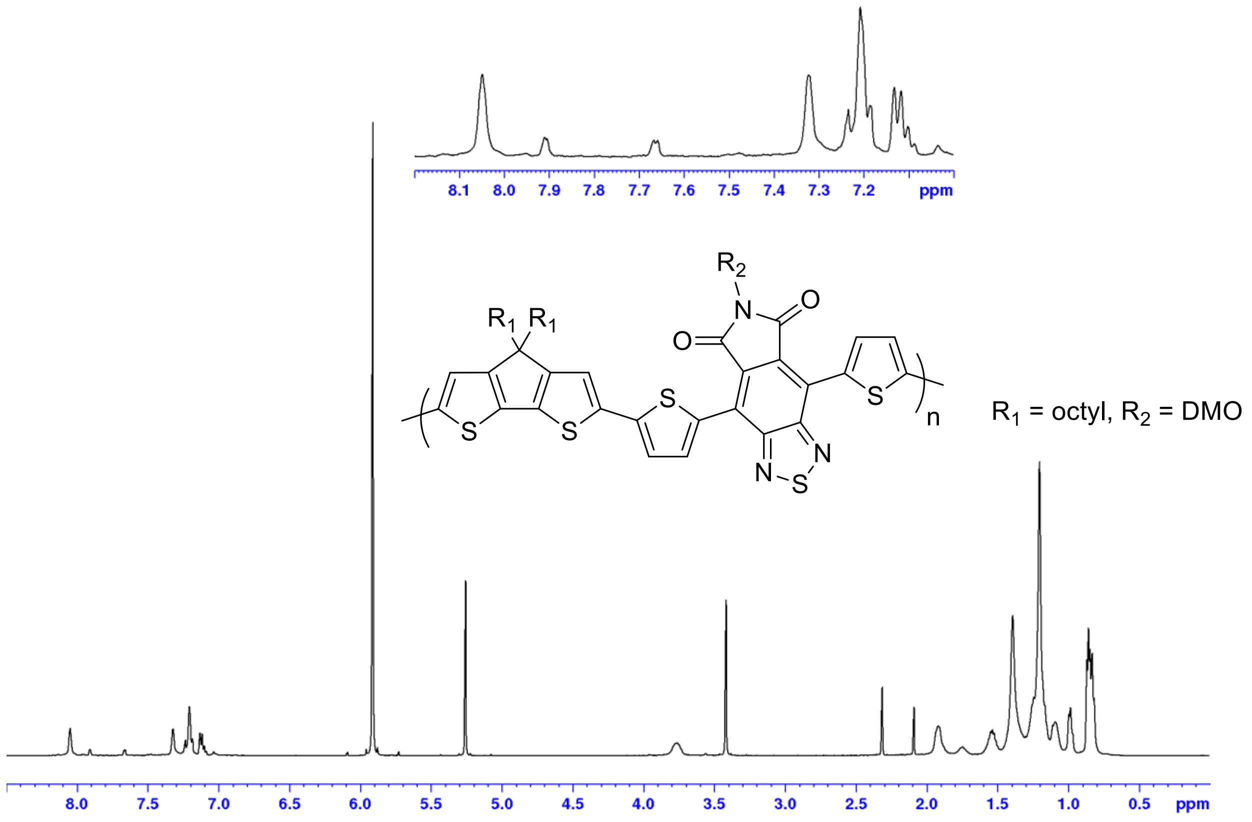

Figure S2. ^{1}H NMR spectrum of PCPDTDTBTDI-8, DMO in $C_2D_2Cl_4$ at 100 ℃.

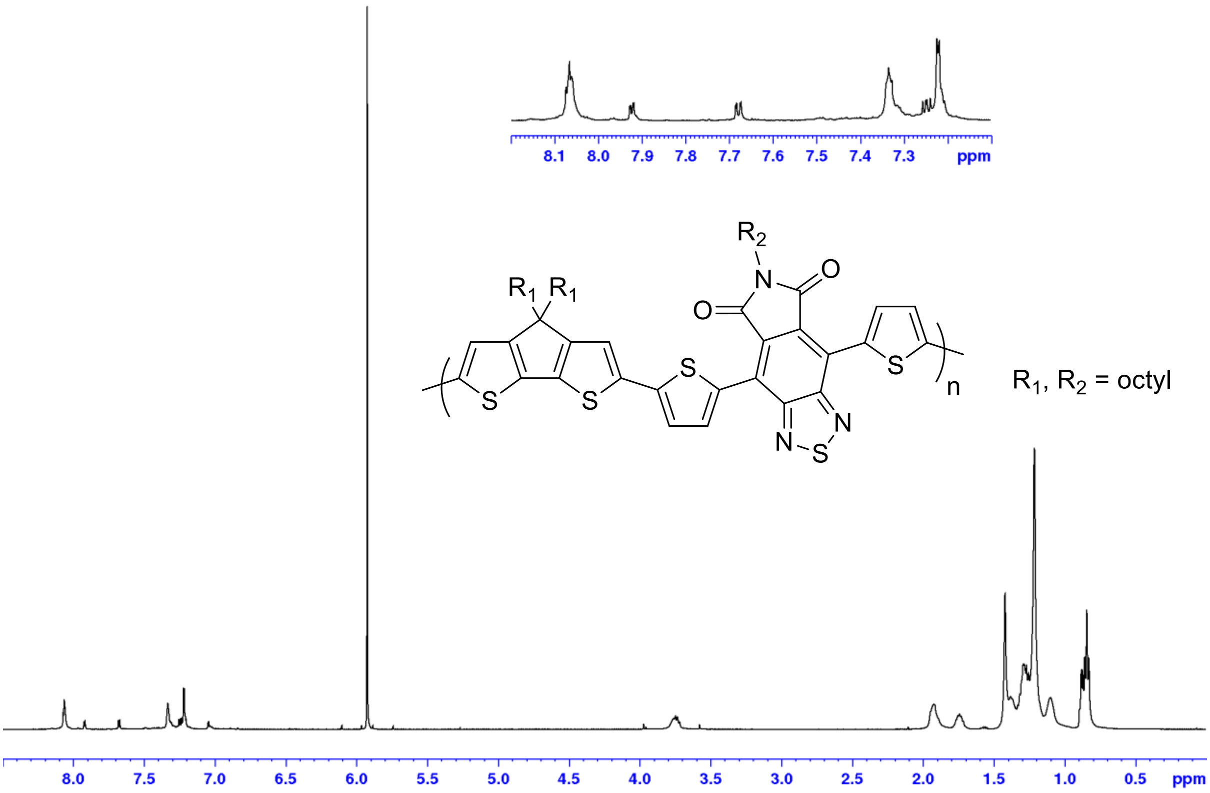

Figure S3. ^{1}H NMR spectrum of PCPDTDTBTDI-8, 8 in $C_2D_2Cl_4$ at 100 ℃.

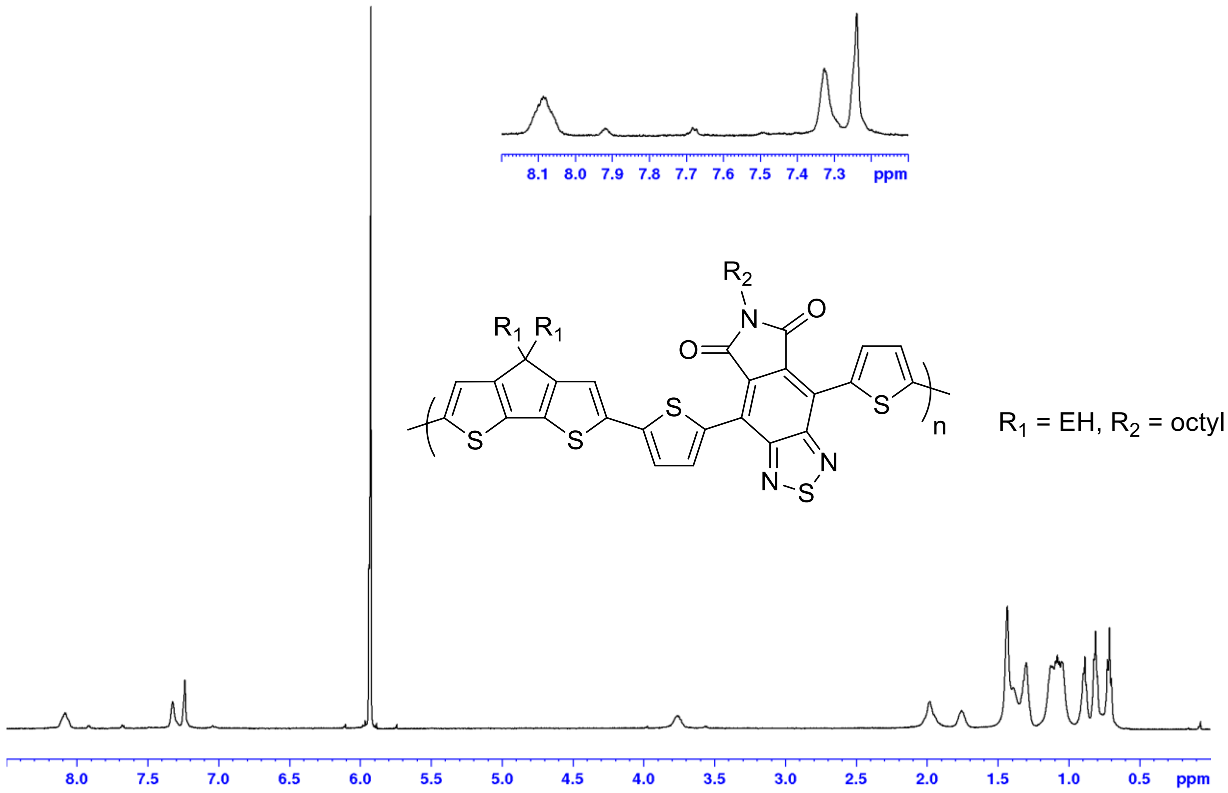

Figure S4. ^1H NMR spectrum of PCPDTDTBTDI-EH, 8 in $C_2D_2Cl_4$ at 100 ℃.
